# Single-cell directional sensing from just a few receptor binding events

**DOI:** 10.1016/j.bpj.2023.06.015

**Published:** 2023-06-24

**Authors:** Andrew J. Bernoff, Alexandra Jilkine, Adrián Navarro Hernández, Alan E. Lindsay

**Affiliations:** 1 Department of Mathematics, Harvey Mudd College, Claremont, California; 2 Department of Applied & Computational Mathematics & Statistics, University of Notre Dame, South Bend, Indiana

## Abstract

Identifying the directionality of signaling sources from noisy input to membrane receptors is an essential task performed by many cell types. A variety of models have been proposed to explain directional sensing in cells. However, many of these require significant computational and memory capacities for the cell. We propose and analyze a simple mechanism in which a cell adopts the direction associated with the first few membrane binding events. This model yields an accurate angular estimate to the source long before steady state is reached in biologically relevant scenarios. Our proposed mechanism allows for reliable estimates of the directionality of external signals using temporal information and assumes minimal computational capacities of the cell.

## Significance

Directional sensing has been observed in many cell types, often at very low concentrations of chemical cues. Cells infer the direction of signaling sources through binding events at membrane bound surface receptors. Since only a few binding events to receptors can trigger a signaling cascade within the cell, we focus on early arrivals at receptor sites. We show that cells can acquire directional information before a steady state of the external chemical signal is reached. We propose a simple mechanism where a cell adopts the direction associated with the first few membrane binding events. This pre-steady-state response is in line with biological observations of cells responding to chemoattractants, or growth of fungi in response to extracellular signals.

## Introduction

Accurately choosing a direction in which to move or grow in response to an external signal is an essential function of a variety of cell types. Examples of such behavior include chemotaxis (cell movement up a chemical gradient) (1,2,3), gradient-directed neuronal cone growth (4), and chemotropism (directed growth toward a pheromone source) (5,6,7). The determination of suitable direction in all these cases must be made from the noisy observations of binding of diffusing external signaling molecules to membrane bound receptors, coupled together with downstream intracellular amplification of the signal (1,7).

Since Berg and Purcell’s pioneering paper on the physics of chemoreception (8), cellular sensitivity to external cues has long been considered through the paradigm of steady-state chemical gradients (9,10,11,12). As the limits of experimental measurements have expanded, effective directional sensing has been observed at lower and lower concentrations. Segall (5) first observed accurate orientation of yeast cells with concentrations of the pheromone α-factor of 67 nM. It was later demonstrated (13) that optimal directional sensing in yeast actually occurs at much lower 5 nM concentrations. Recent experimental studies (14) measured endogenous G-protein-coupled receptor activity in various cell types with high spatial and temporal resolution and established that GPC receptors are capable of responding to femtomolar and attomolar concentrations ($10^{-15}$–$10^{-18}$ M). At such low concentrations, the cell must make decisions from just one or two receptor binding events (14). However, many theoretical models explaining cellular response to chemical cues are based on continuous representations of steady-state concentrations of chemoattractants.

In the context of directional sensing, recovering the source of external stimuli from steady-state receptor fluxes (so-called *splitting probabilities* (15)) is theoretically possible (16,17). Maximum likelihood estimation is a practical method to reconstruct source location from both steady-state (18) and dynamic receptor activity (19). Mechanisms for cellular implementation of maximum likelihood estimation in chemosensing have been proposed (20,21), suggesting biological feasibility; however, such an approach places significant computational requirements on a cell. The cell must know its geometry and the spatial configuration of its receptors, and it must store and integrate a temporal record of binding events. Can a simple mechanism assuming minimal cellular computational capacity provide an accurate directional estimate?

Here, we argue that a cell can reliably acquire accurate directional estimates of signaling sources by considering the earliest receptor binding events. These early events arise from diffusing signaling molecules that closely follow a straight line (shortest) trajectory from the source $x_{0}$ to a receptor (22,23), and hence these paths convey significant directional information to the cell. From a combination of homogenization theory, extreme value theory, and short-time asymptotics for the diffusion equation, we show that an estimate based on the position of the first binding event is highly accurate, provided the source is not too distant. If instead one considers the distribution of the first few arrivals, we find that, while the mean error slowly increases, the variance is significantly reduced.

### Model

**Figure 1:**
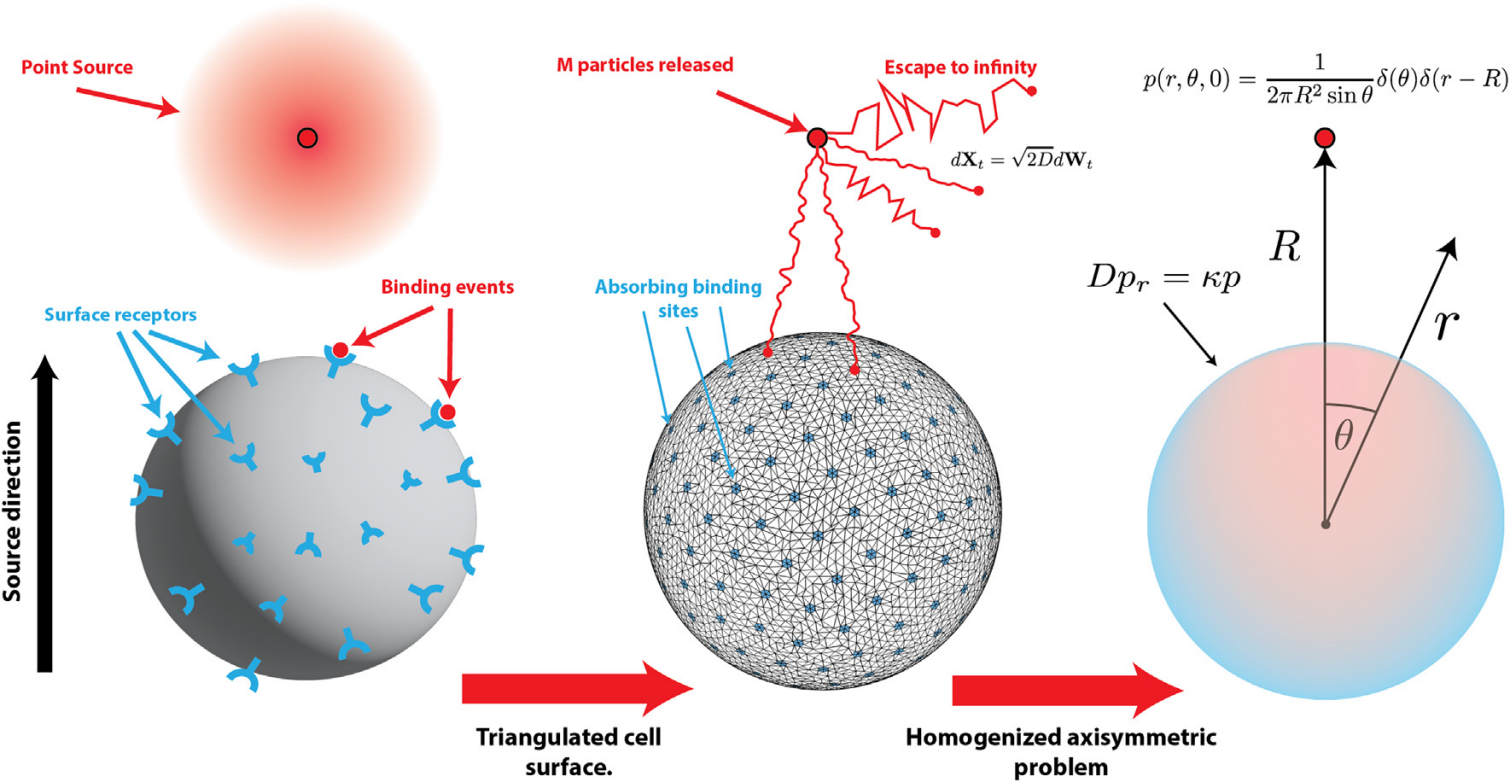
Model. Left: binding events at cell surface receptors give information on source direction. Center: our kinetic Monte Carlo model uses a triangulated cell surface with M diffusing particles released at t=0 from the source at $x_{0}=\left(0,0,R\right)$. Binding events occur when particles reach small circular absorbing sites on the cell surface. Right: axisymmetric continuous formulation as a diffusion equation with a Dirac source at (0,0,R) and homogenized boundary condition $Dp_{r}=\kappa p$ on the cell boundary r=1. To see this figure in color, go online.

We consider the simple conceptual model of Berg-Purcell (8), which continues to serve as a bedrock for understanding receptor activation (24,25,26,27). Let $\Omega \subset R^{3}$ be the unit sphere with N static, circular, nonoverlapping surface receptors of common radius a (cf. Fig. 1). The organization of surface receptors can vary from spatially homogeneous (e.g., GABA (28)) to clustered (e.g., yeast (29)). Clustered (18) and diffusing (30) receptor configurations can be incorporated within this framework; however, we consider here a fixed and spatially uniform configuration of receptors reflective of a cell in a quiescent state. The receptors occupy a surface fraction $\sigma =Na^{2}/4$ and their fixed locations are centered at the Fibonacci spiral points, a well-known and effective covering of the sphere (31,32). To explore the efficacy of using early binding events to infer source direction, we consider M diffusing particles originating at $x_{0}=\left(0,0,R\right)$ for R>1. A number $\left(M_{a}\right)$ of these particles will reach and bind to a receptor while the remaining $\left(M-M_{a}\right)$ will escape to infinity. We simulate this process on a triangulated cell surface and use a particle-based diffusion simulation with kinetic Monte Carlo acceleration (33,34). The arrival times ${\left\{t_{k}\right\}}_{k=1}^{M_{a}}$ are sorted $\left(t_{k}<t_{k+1}\right)$ and the associated binding locations ${\left\{x_{k}\right\}}_{k=1}^{M_{a}}$ recorded. The source is located on the polar axis, with associated unit vector $e_{3}=\left(0,0,1\right)$, and we characterize events via the elevation $z_{k}=\cos\;\theta_{k}=e_{3}\cdot x_{k}$, where $\theta_{k}$ is the angle between the North Pole and the location of the $k^{th}$ binding event. Values $z_{k}=\cos\;\theta_{k}∼1$ indicate alignment with the source, while $z_{k}\;∼\;0$ indicates a uniform distribution over the sphere. Using $M=10^{5}$ initial particles for R=5, we calculate points $\left({\bar{t}}_{n},{\bar{z}}_{n}\right)$ consisting of a running average of $M_{s}$ elevations, specifically

(1) $${\bar{t}}_{n}=\frac{1}{M_{s}}\sum_{k=n}^{n+M_{s}-1}t_{k},\;{\bar{z}}_{n}=\frac{1}{M_{s}}\sum_{k=n}^{n+M_{s}-1}\cos\theta_{k}\text{.}$$

**Figure 2:**
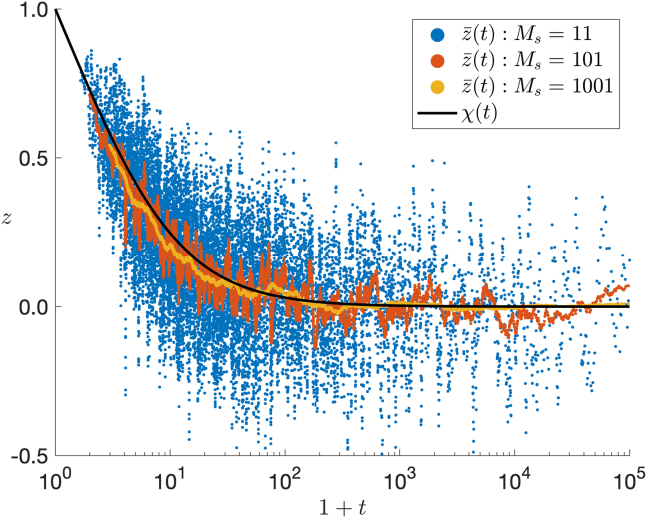
The average elevation coordinate (Eq. 1) of binding events on the membrane for R=5, N=201, D=1, σ=0.05, and $M=10^{5}$. Variance reduction is achieved by increasing the number of binding events, $M_{s}$ in the running average. The parameter from Eq. 3 is κ≈3.36, which yields the homogenized theoretical prediction χ(t) from Eq. 7. To see this figure in color, go online.

In Fig. 2, we plot Eq. 1 for values $M_{s}=\left\{11,101,1001\right\}$. At short times we observe that elevations $\bar{z}∼1$, corresponding to binding events aligned in the source direction. At later times, particles gradually lose information on their initial position and binding events occur uniformly on the surface ($\bar{z}\;∼\;0$ as t→∞). Increasing the number of averaging points $M_{s}$ reduces the variance. This simulation suggests that, at short times, the directions associated with binding events give an estimate strongly biased toward the source. How short is “short enough” and how accurate is such an estimate? To answer this, we analyze a homogenized partial differential equation (PDE) model.

### Homogenization

Boundary homogenization theory (19,34,35,36,37,38,39) posits that the complex configuration of surface receptors and associated mixed boundary conditions can be replaced by the Robin condition, $D\partial_{\nu }p=\kappa p$ on ∂Ω, where $\partial_{\nu }\equiv \hat{n}\cdot \nabla$ is the normal derivative. For $x_{0}=\left(0,0,R\right)$, the axisymmetric particle density p(r,θ,t;R) solves the initial boundary value problem

(2a) $$\frac{\partial p}{\partial t}=D\left[\frac{\partial^{2}p}{\partial r^{2}}+\frac{2}{r}\frac{\partial p}{\partial r}+\frac{1}{r^{2}\sin\theta }\frac{\partial^{2}p}{\partial \theta^{2}}\right],\;t>0,\;r>1,\;0<\theta <\pi \text{;}$$

(2b) $$D\partial_{r}\;p=\kappa p,\;t>0,\;r=1,\;0<\theta <\pi \text{;}$$

(2c) $$p=\frac{1}{2\pi R^{2}\sin\theta }\delta \left(r-R\right)\delta \left(\theta \right),\;t=0,\;r>1,\;0<\theta <\pi$$

**Figure 3:**
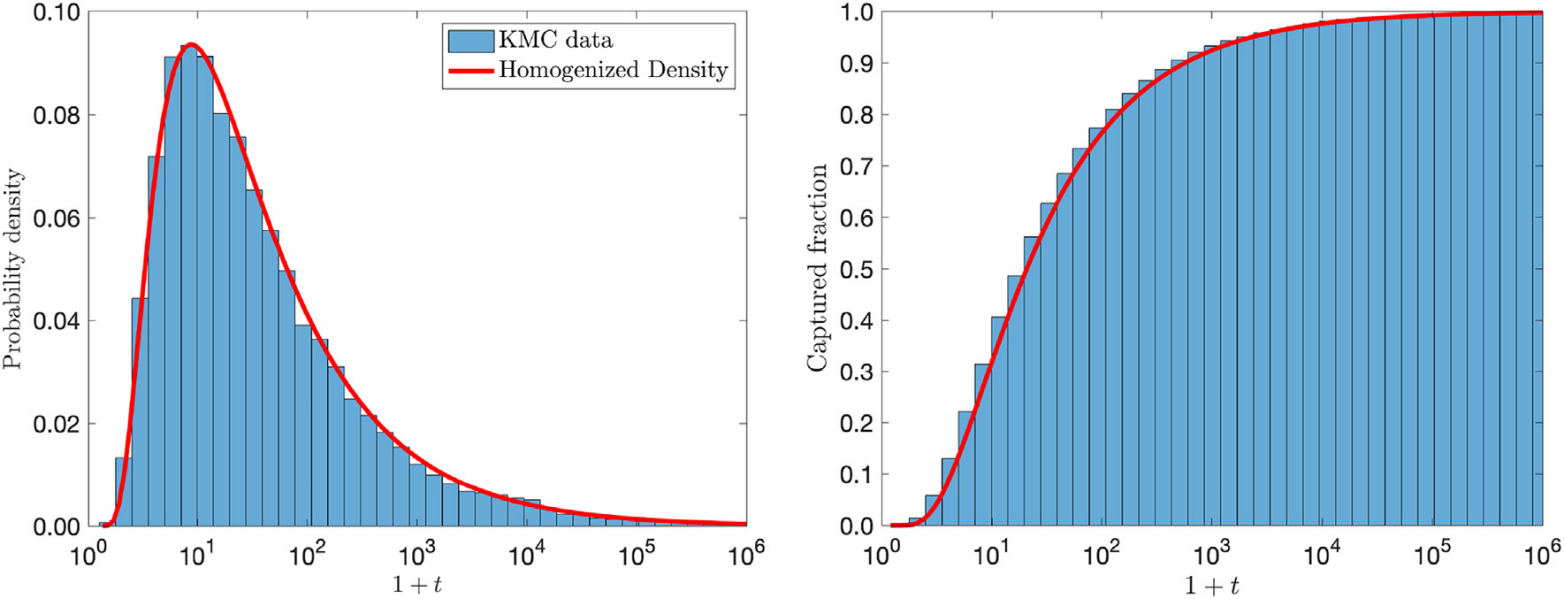
Validation of boundary homogenization for R=5, N=201, σ=0.05, D=1, and $M=10^{5}$. The formula Eq. 3 gives κ≈3.36. The full distribution of arrival times (*left*) and the cumulative density function of captured particles (*right*) from Monte Carlo (*histograms*) and homogenization theory Eq. 5 (*solid red*). To see this figure in color, go online.

In our recent work (19), we considered a circular two-dimensional cell and established that the correct homogenization of the full time-dependent dynamics is given by the established homogenization of the steady-state problem (40,41). Here, we conjecture and numerically verify in Fig. 3 that a similar result holds for a spherical cell. Specifically, for the case of uniformly distributed receptors with combined surface fraction σ≪1, we posit that the density can be recovered by applying the Robin condition (Eq. 2b) with

(3) $$\kappa =\frac{4\sigma }{\pi a}{\left[1-\frac{4}{\pi }\sqrt{\sigma }+\frac{a}{\pi }\log\left(4\sqrt{\sigma }e^{-\frac{1}{2}}\right)+\frac{a^{2}}{2\pi \sqrt{\sigma }}\right]}^{-1}\text{,}$$

where Eq. 3 is the homogenization parameter derived from the steady-state flux (38,42,43). The solution of Eq. 2 is separable and available in terms of spherical Bessel expansions. We find (cf. supporting material) the surface flux $J=D\partial_{r}p{|}_{r=1}$ to be

(4a) $$J\left(\theta ,t\right)=\frac{1}{2\pi }\sum_{n=0}^{\infty }\frac{2n+1}{2}\psi_{n}\left(t;R\right)P_{n}\left(\cos\;\theta \right)\text{.}$$

Here, $P_{n}\left(z\right)$ are the Legendre polynomials. The coefficients are determined through Laplace transform ${\hat{\psi }}_{n}\left(s;R\right)=\int_{t=0}^{\infty }\psi_{n}\left(t;R\right)e^{-st}dt$ and are given by

(4b) $${\hat{\psi }}_{n}\left(s;R\right)=\frac{k_{n}\left(cR\right)}{k_{n}\left(c\right)-c\frac{D}{\kappa }k_{n}^{'}\left(c\right)},\;c=\sqrt{\frac{s}{D}}\text{.}$$

Here, $k_{n}\left(z\right)$ is the modified spherical Bessel function of the second kind. The total flux $\rho \left(t\right)=2\pi \int_{\theta =0}^{\pi }J\left(\theta ,t\right)\sin\;\theta d\theta$ is

(5) $$\rho \left(t\right)=\frac{\kappa }{R}e^{\frac{-{\left(R-1\right)}^{2}}{4Dt}}\left[\frac{1}{\sqrt{\pi Dt}}-\left(\frac{\kappa }{D}+1\right)\text{erfcx}\left(\beta \right)\right]\text{,}$$

where $\beta =\left(R-1\right)/\sqrt{4Dt}+\left(\frac{\kappa }{D}+1\right)\sqrt{Dt}$ and $\text{erfcx}\left(z\right)=\frac{2}{\sqrt{\pi }}e^{z^{2}}\int_{z}^{\infty }e^{-t^{2}}\;dt$ is the scaled complementary error function. We remark that

(6) $$\int_{0}^{\infty }\rho \left(t\right)dt=\frac{1}{\left(1+\frac{D}{\kappa }\right)R}\text{,}$$

so that the probability of capture is not unity, but inversely proportional to the initial distance to the sphere. In fact, this result provides the expected fraction of binding events $E\left[M_{a}\right]=M/\left[\left(1+D/\kappa \right)R\right]$. In Fig. 3, we demonstrate with comparison to Monte Carlo simulations that the homogenized solution accurately predicts the capture rate. At what time do binding events cease to convey information on the source direction? The answer can be gleaned by considering from Eq. 4a the average elevation of events at time t given by

(7) $$\chi \left(t\right)=\frac{\int_{\theta =0}^{\pi }\;\cos\;\theta J\left(\theta ,t\right)\sin\;\theta \;d\theta }{\int_{\theta =0}^{\pi }J\left(\theta ,t\right)\sin\;\theta \;d\theta }=\frac{\psi_{1}\left(t\right)}{\psi_{0}\left(t\right)}\text{.}$$

At large times the function χ(t) tends to zero as the surface flux becomes radially symmetric and consequently arriving particles yield no information on the source direction. In Fig. 2 we see that χ(t) agrees well with numerical results and thus predicts the time-dependent bias of the surface flux toward the source. The close agreement in Fig. 2 provides an additional nontrivial validation of the boundary homogenization approach.

### Extreme arrivals

We have observed that averaging of binding events at short times gives an estimate biased toward the source. We now focus on calculating the distribution of the times and locations of the earliest binding events, usually referred to as *extreme arrivals*. Our previous study (19) explored dynamics two-dimensionally with characterization of equilibrium dynamics and short-time fluxes. Here, we consider the distribution of the $k^{th}$ arrival to the sphere in three dimensions. Specifically, consider M particles released from $x_{0}=\left(0,0,R\right)\text{,}$ which results in binding events at times ${\left\{t_{k}\right\}}_{k=1}^{M_{a}}$ with $t_{k}<t_{k+1}$. Lawley (23) determined for M≫1 that $t_{k}$ follows the Gumbel distribution with mean and variance determined by the limiting behavior of the survival probability $S\left(t\right)=P\left[t_{1}<t\right]$ as $t\to 0^{+}$. We calculate (cf. supporting material) that $S\left(t\right)∼1-h\left(t\right)\exp\left[-\frac{{\left(R-1\right)}^{2}}{4Dt}\right]$ as $t\to 0^{+}$ where

(8) $$h\left(t\right)=\sqrt{\frac{D}{\pi }}\frac{4\kappa }{R\left(R-1\right)}\left[\frac{t^{\frac{3}{2}}}{\left(R-1\right)+2\kappa t}\right]\left[1+O\left(Dt\right)\right]\text{.}$$

Applying ((23), Prop 3, and Thm 4.), we have that

(9) $$\frac{t_{k}-b_{M}}{a_{M}}\to X_{k},\;\text{as}\;M\to \infty \text{,}$$

where the density of $X_{k}$ is given by

(10) $$P\left[X_{k}=x\right]=\frac{e^{kx-e^{x}}}{\left(k-1\right)!}\text{.}$$

The centering and scaling parameters, $b_{M}$ and $a_{M}$, respectively, are given by the relations

(11) $$S\left(b_{M}\right)=1-M^{-1},\;a_{M}=-{\left(MS^{'}\left(b_{M}\right)\right)}^{-1}\text{.}$$

Eq. 11 is amenable to numerical and asymptotic solution for M≫1 (cf. supporting material). At leading order

(12) $$b_{M}∼\frac{{\left(R-1\right)}^{2}}{6D\nu },\;\nu =\log\;{\left[\sqrt{\frac{2}{27\pi }}\frac{M\kappa \left(R-1\right)}{RD}\right]}^{\frac{2}{3}}\text{.}$$

We calculate the distribution of the angle $\theta_{k}$ to be

(13) $$\begin{array}{cc} P\left[\theta_{k}=\eta \right] & =\int_{\tau =0}^{\infty }P\left[\theta =\eta |t=\tau \right]P\left[t_{k}=\tau \right]d\tau \\ & \approx \frac{J\left[\theta =\eta |t=b_{M}+a_{M}\log k\right]}{\rho \left(b_{M}+a_{M}\log k\right)}\text{.} \end{array}$$

The approximation leading to Eq. 13 is based on Laplace’s method with evaluation at $\text{Mode}\left[X_{k}\right]=b_{M}+a_{M}\;\log\;k$, the value around which the distribution is highly peaked. In terms of z=cosθ, we determine (cf. supporting material) as $t\to 0^{+}$ that

(14a) $$J\left(z,t\right)∼\frac{\rho \left(t\right)}{\lambda }e^{-\frac{1-z}{\lambda }},\;\lambda =\frac{2Dt}{R}\text{;}$$

(14b) $$\rho \left(t\right)∼\frac{\kappa }{R}\;\frac{e^{-\frac{{\left(R-1\right)}^{2}}{4Dt}}}{\sqrt{\pi Dt}}\left[\frac{R-1}{\left(R-1\right)+2\kappa t}\right]\text{,}$$

Combining Eq. 13 and Eq. 14, we conclude for M≫1 that the elevation from the North Pole of the $k^{\text{th}}$ binding event has exponential distribution $\left(1-z_{k}\right)\;∼\;\text{Exp}\left(\lambda_{k}^{-1}\right)$ with

(15) $$\lambda_{k}=\frac{2D}{R}\left[b_{M}+a_{M}\;\log\;k\right]\text{.}$$

Smaller values of $\lambda_{k}$ are associated with a more accurate directional estimate. Since $\lambda_{k}<\lambda_{k+1}$, the directional information of the first event (k=1) yields the lowest error and smallest variance compared with subsequent events (k>1). Further applying Eq. 12 in the limit as M→∞, we obtain

(16) $$\lambda_{1}∼\frac{{\left(R-1\right)}^{2}}{3R\nu }∼\frac{{\left(R-1\right)}^{2}}{2R\;\log\;M}\text{.}$$

**Figure 4:**
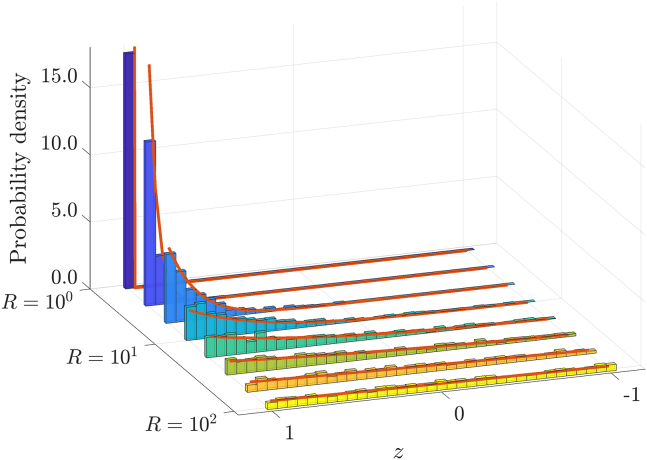
Distribution of locations for first binding event $z_{1}=\cos\;\theta_{1}$ at eight equally log-spaced points from $R=10^{0}$ to $R=10^{2}$. Parameters κ=3.36, D=1, and $M=10^{5}$. Histograms based on 1000 independent realizations with theoretical predictions (Eq. 4) in close agreement (*solid red*). To see this figure in color, go online.

The form of Eq. 16 reveals that lower errors are associated with large M and small values of (R−1). Hence, we conclude that a cell can make an accurate estimate of a source direction from just a single binding event, provided (R−1) is not too large. In Fig. 4 we show numerical validation of the distribution of $z_{1}$ as predicted by Eq. 14 based on 1000 independent realizations and parameter values κ=3.34, D=1, and $M=10^{5}$.

### Multiple binding events

Could an improvement to this simple estimate be formed by considering several early binding events? The average elevation of the first K events is given by the variable $Z_{K}=\frac{1}{K}\sum_{i=1}^{K}z_{i}$. We determine that $1-Z_{K}∼\;\text{Hypo}\left(\frac{K}{\lambda_{1}},\ldots ,\frac{K}{\lambda_{K}}\right)$ where a *hypoexponential* variable $Z\;∼\;\text{Hypo}\left(\alpha_{1},\ldots ,\alpha_{K}\right)$ has density

(17) $$P\left[Z=z\right]=\sum_{i=1}^{K}w_{i}\alpha_{i}e^{-\alpha_{i}z},\;w_{i}=\prod_{\begin{array}{l} j=1\\ j\neq i \end{array}}^{K}\frac{\alpha_{j}}{\alpha_{j}-\alpha_{i}}\text{.}$$

The mean and variance of the error $1-Z_{K}$ are calculated to be

(18a) $$E\left[1-Z_{K}\right]=\sum_{i=1}^{K}\frac{\lambda_{i}}{K}=\frac{2D}{R}\left(b_{M}+\frac{a_{M}}{K}\log\;K!\right)\text{,}$$

(18b) $$\text{Var}\left[1-Z_{K}\right]=\sum_{i=1}^{K}\frac{\lambda_{i}^{2}}{K^{2}}=\frac{4D^{2}}{R^{2}K^{2}}\sum_{i=1}^{K}{\left(b_{M}+a_{M}\;\log\;i\right)}^{2}\text{.}$$

**Figure 5:**
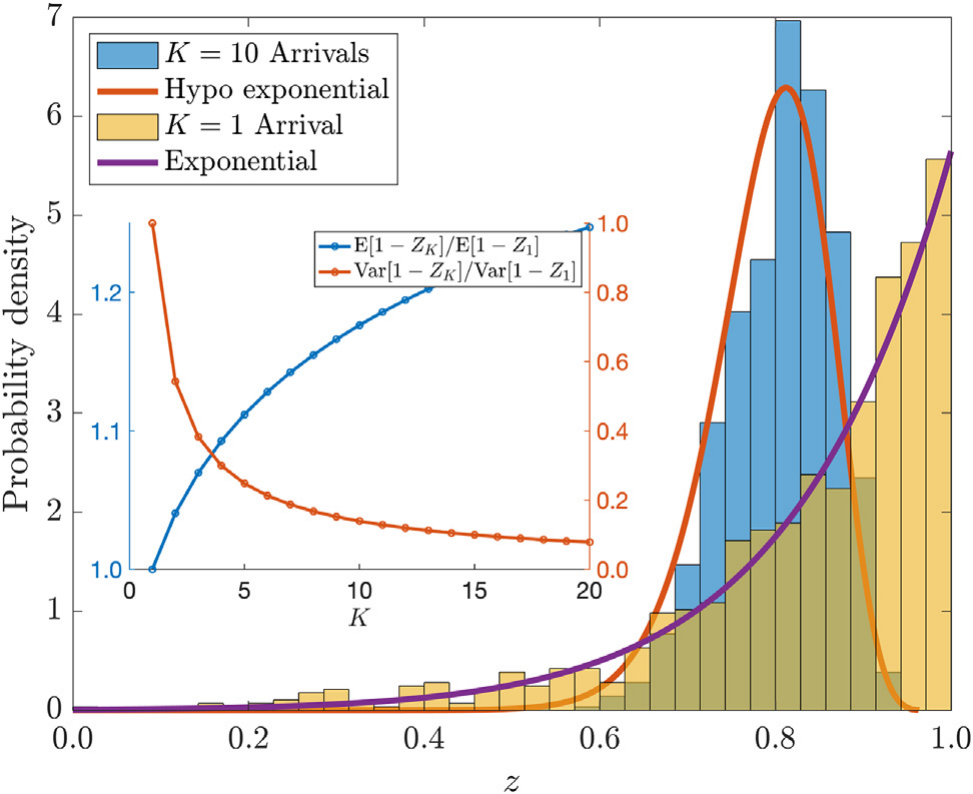
Demonstration of variance reduction from averaging several binding events with parameters R=4.4, κ=3.36, D=1, and $M=10^{5}$. Outer figure: distributions of $Z_{K}$ for K=10 and K=1 based on 1000 realizations. Inset: relative changes in mean and variance of estimate for a range of K values. As K increase the mean error (Eq. 18a) increases slowly while the variance (Eq. 18b) can be reduced substantially. To see this figure in color, go online.

We hence conclude from Eq. 18 that taking the average of K early arrivals results in a slight precession of the mean away from the correct value $Z_{K}=1$, but yields a smaller variance, therefore generating a tighter distribution. We demonstrate this effect in Fig. 5 for the parameters R=4.4, $M=10^{5}$, D=1, κ=3.36 with data shown for 1000 realizations. The distribution of arrivals from the first (K=1) arrival is exponential and the average of the first K=10 arrivals is hypoexponential. As demonstrated in the inset of Fig. 5, an increase in the number of events K averaged yields a slight deterioration in the average error but a large reduction in the variance. For example, averaging over just K=5 events increases the mean error by approximately 11% compared with the first arrival, but decreases the error variance by roughly a factor of 4. The trade-off between the error’s increasing mean (Eq. 18a) and decreasing variance (Eq. 18b) as a function of K suggests that there may be an optimal number of binding events. In the supporting material we explore this trade-off.

### Application to chemosensing

Budding yeast *Saccharomyces cerevisiae* is a well-studied model system for chemosensing. In *S. cerevisiae* polarized growth toward a mating partner is guided by diffusible chemoattractants, such as α-factor (7). Experimental studies have reported that yeast cells are capable of sensing gradients as shallow as 0.1 nM/μm (13,44) and do so over 1000-fold range of α-factor concentrations. Measured cosθ values of yeast mating projections (13), analogous to our estimated chemoattractant coordinate (cf. Fig. 2), show gradient detection accuracy was maximized for cosθ≈0.8 in 0−5 nM concentration gradient. Accuracy decreases at higher α-factor concentrations and shallower gradients, but the cells are able to sense direction over a broad range of concentrations and gradient slopes, even at concentrations as high as 1 μM, with the typical cosθ≈0.6, and in a uniform gradient cosθ≈0 as expected. It is important to note that microfluidic experiments done by (13) used a steady-state gradient, while our model and simulations use a point source, and are a better representations of experiments by (5), who first demonstrated that yeast cells could sense a chemoattractant source emitted using a micropipette and form a projection toward the source.

The binding dynamics of α-factor to its receptor are known to be slow. At concentrations near the dissociation constant ($K_{d}\approx 5$ nM), binding takes about 20 min to reach 90% of the equilibrium level (45). However, the yeast cell starts responding to the chemoattractant by assembling a polarity site within minutes, and later potentially moves the polarity site to track the chemotactic source on a longer timescale. This supports our hypothesis that chemosensing starts long before a steady-state gradient may be reached. As noted by (45), extracting information pre-equilibrium can overcome both the noise issue and receptor saturation limits, and expand the input dynamic range of chemoattractant to which budding yeast cells can respond.

Another example of directional sensing occurs in chemotactic cells such as *Dictyostelium discoideum* and neutrophils, where, when a chemoattractant is released from a pipette, a response is observed in 2–5 s (1,46,47). Thus, the first step in chemosensing must occur on this timescale. Our predictions of extreme statistics are of roughly the same order of magnitude as those observed experimentally for *Dictyostelium* and provide a minimum time for a cell to respond to a diffusive signal.

Chemosensing involves the cell surface receptors binding to extracellular diffusing molecules. What is the typical receptor number N on different types of cells that need to respond to external chemical sources? Physiological receptor numbers vary considerably: $N\approx 10^{2}-10^{3}$ in receptors in neural cone growth (4,28), $N\approx 10^{4}$ in budding yeast (48), and $N\approx 10^{4}-10^{5}$ in lymphocytes (49). Activation of these receptors then induces the production of second-messenger molecules that then transduce the external signal to downstream signaling cascades within a cell. In our model, we considered chemotactic source molecules diffusing in a three-dimensional region and interacting with receptors uniformly embedded on the surface of a single spherical cell. We have not considered the effects of receptor clustering, extracellular ligand unbinding from the receptor once a binding event occurs, receptor internalization, and the duration of receptor being in a bound state to the ligand. All these effects are potentially important in different biological contexts (36,50). For example, ratiometric sensing, by considering the ratio of active to inactive receptors, compensates for uneven receptor density in *S. cerevisiae* and allows more accurate gradient detection (6,7). At steady state, the fraction of bound receptors depends on both the external chemical concentration and the binding dissociation constant $K_{d}$. Interestingly, (50) uses a particle-based reaction-diffusion model of *S. cerevisiae* ligand-receptor dynamics and reports that neither time-averaging nor receptor endocytosis significantly improves the cell’s accuracy in detecting gradients over timescales associated with the initiation of polarized growth in yeast. Our proposed chemo-detection mechanism does not consider the putative positive feedback loops downstream of receptors that allow cells to fine tune its chemosensing machinery on a longer timescale (29,51,52). However, this means that there are no unknown parameters to fit in our phenomenological model, which is often not the case for more detailed mechanistic models.

## Discussion

In this paper we propose and analyze a simple method for directional sensing based on the estimates formed by the first few binding events of signaling molecules to membrane receptors. In contrast to limits imposed on gradient sensing at equilibrium, we shown here that, long before equilibrium is attained, these early receptor binding events confer sufficient information to accurately estimate the source direction. Among M events, we characterize the distribution associated with the average of the first K arrivals for 1≤K≪M as hypoexponential. A directional estimate based on just the first binding is accurate, provided the source is not too distant. Since we focus on the initial few binding events, the issue of receptor saturation, which can occur at high chemoattractant concentrations and can be potentially alleviated by receptor recycling, is not an issue on the timescales that we are considering. From our analysis, we expect that signal strength (M) and source distance (R) are the main factors that determine the efficacy of extreme statistics in directional sensing. Factors, such as receptor binding/unbinding, can be thought of as modulating κ, which our analysis shows to be a lower-order effect (36). Hence, when one is interested only in the distribution of the first few binding events, mean receptor occupancy by the ligand does not play a large role.

Gradient sensing strategies fall into two major categories: temporal and spatial. Temporal sensing mechanisms, thought to be used by bacteria, involve an organism moving and sampling the concentration of chemoattractant in its environment. Spatial sensing mechanisms, in which the organism compares receptor occupancy difference across the cell body, are thought to be used by larger eukaryotic chemotactic cells (1,9). The fact that yeast cells are not motile has been used to suggest that they also use a spatial sensing mechanism, despite being smaller (4 μm in diameter) than most eukaryotic cells (7). However, as we show here, an immobile cell can still use temporal information to help it detect a chemotactic source before a steady state is reached. Our model has some similarities to the “first hit” model proposed by (53). In that work, initial activation of receptors activates the side of the cell closest to the stimulus and triggers a rapid inhibitory response that spreads across the cell and prevents the posterior from responding. When the gradient is repositioned, there is again an initial contact and the direction of the response is reset. Most modeling literature on chemosensing in eukaryotes uses deterministic models and assumes that the signal from the chemotactic source is at equilibrium (54). Here, we argue that immobile cells can actually acquire a lot of information from the time-dependent problem. Chemotactic cells can orient toward a micropipette source on a very rapid timescale, and this process also occurs in immobilized cells that cannot undergo cell shape change (1). Cellular response toward the source of chemical cues has been also been observed only a few seconds after exposure to a chemoattractant (47). It would be interesting to combine the stochastic direction-sensing mechanism we propose here to some of the proposed models for gradient amplification and cell polarization downstream of the receptors (11,54,55,56,57) and predict the frequency of reorientation of the cell to a changing source position.

Sensing of multiple sources with general spatial and temporal distributions is a natural extension of this work. The linearity of the underlying problem allows for superposition of the solutions developed here. In such a scenario, the surface flux J(θ,t) would exhibit multiple peaks in space and time whose structure would need to be resolved. Similar recovery analysis has been accomplished using Fourier methods in the context of defect localization (58). In addition, it would also be interesting to consider the effect of nonspherical cell geometry on chemosensing for cell types such as *Dictyostelium* and neutrophils.

Finally, directional sensing is often performed in a group of cells, with putative feedback from other cells (59,60,61,62,63). Another important biological problem, where multiple cells need to determine their position within a tissue due to an external chemical gradient of a *morphogen* (64), has traditionally assumed due to the timescale of development that the morphogen gradient is at steady state (65,66). However, there is increasing biological evidence that some morphogen gradients may actually start being interpreted before reaching steady state (67,68,69). Theoretical work suggests that pre-steady-state measurements of the morphogen gradient may reduce the effects of stochastic fluctuations on determining spatial boundaries in tissue (70). It would be interesting to revisit our proposed chemosensing mechanism for a group of cells.

## Author contributions

A.E.L. and A.B. designed the research, performed research, and wrote the paper. A.J. analyzed the data and wrote the paper. A.N.H. analyzed the data and performed the research.

## Supporting material

Document S1. Figures S1

Document S2. Article plus supporting material
