# Supplementary material for: Single-cell directional sensing from just a few receptor binding events
Source: Biophys J. 2023 Jun 24;122(15):3108–16. doi: 10.1016/j.bpj.2023.06.015 (PMC10432224; doi:10.1016/j.bpj.2023.06.015)
Supplement: Document S1. Figures S1 [file mmc1.pdf]

Biophysical Journal, Volume *122*

Supplemental information

Single-cell directional sensing from just a few receptor binding events

Andrew J. Bernoff, Alexandra Jilkine, Adrián Navarro Hernández, and Alan E. Lindsay

Manuscript submitted to **Biophysical** *Journal*
Article

Supplemental material to Single cell directional sensing from just a few receptor binding events.

Andrew J. Bernoff[a], Alexandra Jilkine[b], Adrián Navarro Hernández[b], and Alan E. Lindsay[b]

[a]Department of Mathematics, Harvey Mudd College, CA, 91711, USA.
[b]Department of Applied & Computational Mathematics & Statistics, University of Notre Dame, South Bend, IN, 46556, USA
[*]Correspondence: a.lindsay@nd.edu

1 SPHERICAL ARRIVAL: HOMOGENIZED BC

We wish to solve for the spherical arrival time $p(r,\theta,t)$ which satisfies the axisymmetric three dimensional diffusion problem

$$\frac{\partial p}{\partial t} = D\left[\frac{1}{r^2}\frac{\partial}{\partial r}\left(r^2\frac{\partial p}{\partial r}\right) + \frac{1}{r^2\sin\theta}\frac{\partial}{\partial\theta}\left(\sin\theta\frac{\partial p}{\partial\theta}\right)\right], \qquad r>1, \quad \theta\in(0,\pi) \tag{1a}$$

$$Dp_r(1,\theta,t) = \kappa p(1,\theta,t), \qquad \theta\in(0,\pi); \tag{1b}$$

$$p(r,\theta,0) = \frac{1}{2\pi R^2\sin\theta}\delta(r-R)\delta(\theta), \qquad r>1, \quad \theta\in(0,\pi), \tag{1c}$$

for which we wish to compute the surface flux density $\mathcal{J}(\theta,t) = Dp_r(1,\theta,t)$. A solution to (1) is sought via the Laplace transform, $\hat{p}(r,\theta) = \int_0^\infty e^{-st}p(r,\theta,t)dt$ where $\hat{p}(r,\theta)$ solves the modified Helmholtz equation,

$$D\left[\frac{1}{r^2}\frac{\partial}{\partial r}\left(r^2\frac{\partial \hat{p}}{\partial r}\right) + \frac{1}{r^2\sin\theta}\frac{\partial}{\partial\theta}\left(\sin\theta\frac{\partial \hat{p}}{\partial\theta}\right)\right] - s\hat{p} = \frac{-1}{2\pi R^2\sin\theta}\delta(r-R)\delta(\theta) \quad r>1, \quad \theta\in(0,\pi); \tag{2a}$$

$$D\hat{p}_r(1,\theta) = \kappa\hat{p}(1,\theta), \quad \theta\in(0,\pi). \tag{2b}$$

for which the Laplace transform of the surface flux density satisfies

$$\widehat{\mathcal{J}}(\theta,s) = \int_0^\infty e^{-st}\left[Dp_r(1,\theta,t)\right]dt = D\hat{p}_r(1,\theta).$$

We construct a separable series solution of (2) which is finite as $r\to\infty$, satisfies $D\hat{p}_r(1,\theta) = \kappa\hat{p}(1,\theta)$, and is continuous at $r=R$,

$$\hat{p}(r,\theta) = \begin{cases} \displaystyle\sum_{n=0}^{\infty} A_n\left[i_n(cr) - \frac{cDi_n'(c)-\kappa i_n(c)}{cDk_n'(c)-\kappa k_n(c)}k_n(cr)\right]P_n(\cos\theta), & r<R; \\ \displaystyle\sum_{n=0}^{\infty} A_n\left[\frac{i_n(cR)}{k_n(cR)} - \frac{cDi_n'(c)-\kappa i_n(c)}{cDk_n'(c)-\kappa k_n(c)}\right]k_n(cr)P_n(\cos\theta), & r>R, \end{cases} \tag{3}$$

where $c=\sqrt{s/D}$ and $k_n'(x)$, $i_n'(x)$ are derivatives of the modified spherical Bessel functions. The constants A_n are fixed by incorporating the Dirac source on the right hand side of (2a). The jump condition is

$$2\pi cDR^2A_n\left[\frac{i_n(cR)}{k_n(cR)}k_n'(cR) - i_n'(cR)\right] = -\frac{(2n+1)}{2}. \tag{4}$$

Solving yields

$$A_n = \frac{1}{2\pi D}\frac{(2n+1)}{2}c\,k_n(cR),$$

where we have used the Wronskian identity $i_n'(z)k_n(z) - i_n(z)k_n'(z) = z^{-2}$. Now returning to $c = \sqrt{s/D}$, the Laplace transform of flux through the spherical surface is then

$$\begin{aligned}
\widehat{\mathcal{J}}(\theta, s) &= D\hat{p}_r(1, \theta, s) \\
&= cD \sum_{n=0}^{\infty} A_n \left[i_n'(c) - \frac{cDi_n'(c) - \kappa\, i_n(c)}{cDk_n'(c) - \kappa\, k_n(c)} k_n'(c) \right] P_n(\cos\theta), \\
&= \frac{1}{2\pi} \sum_{n=0}^{\infty} \frac{(2n+1)}{2} \left[\frac{k_n(cR)/k_n'(c)}{k_n(c)/k_n'(c) - cD/\kappa} \right] P_n(\cos\theta). \\
&= \frac{1}{2\pi} \sum_{n=0}^{\infty} \frac{(2n+1)}{2} \psi_n(c) P_n(\cos\theta), \qquad \psi_n(c) = \frac{k_n(cR)/k_n'(c)}{k_n(c)/k_n'(c) - cD/\kappa}. \qquad (5)
\end{aligned}$$

The Laplace transform of the total flux is

$$\begin{aligned}
\widehat{\rho}(s) &= 2\pi \int_0^{\pi} \widehat{\mathcal{J}}(\theta, s)\, \sin\theta\, d\theta \\
&= \int_0^{\pi} \sum_{n=0}^{\infty} \frac{(2n+1)}{2} \psi_n(c) P_n(\cos\theta)\, \sin\theta\, d\theta \\
&= \sum_{n=0}^{\infty} \frac{(2n+1)}{2} \psi_n(c) \int_{-1}^{1} P_n(x)\, dx \\
&= \psi_0(c) = \frac{k_0(cR)/k_0'(c)}{k_0(c)/k_0'(c) - cD/\kappa} = \frac{e^{-(R-1)\sqrt{s/D}}}{R[1 + (D/\kappa)\cdot(1 + \sqrt{s/D})]},
\end{aligned}$$

where we've used the zero mean property of the Legendre polynomials. The inverse Laplace transform of this quantity is the distribution of arrival times at the sphere,

$$\rho(t) = \frac{\kappa}{R} e^{-\frac{(R-1)^2}{4Dt}} \left[\frac{1}{\sqrt{\pi D t}} - \operatorname{erfc}(\beta)\, e^{\beta^2}\, (\kappa/D + 1) \right]. \qquad (6)$$

where $\beta = \frac{R-1}{2\sqrt{Dt}} + (\kappa/D + 1)\sqrt{Dt}$.

The CDF of this distribution can then be calculated as

$$F(t) = \int_0^t \rho(\tau)\, d\tau = \frac{1}{(1 + D/\kappa)\, R} \left[\operatorname{erfc}\left(\frac{R-1}{2\sqrt{Dt}} \right) - \operatorname{erfc}(\beta)\, e^{\beta^2} e^{-\frac{(R-1)^2}{4Dt}} \right]. \qquad (7)$$

We remark that

$$\int_0^{\infty} \rho(t)\, dt = \lim_{t\to\infty} F(t) = \frac{1}{(1 + D/\kappa)R}$$

so that the probability of capture is not unity, but inversely proportional to the initial distance to the sphere.

1.1 Short time asymptotics via the method of moments

In this section we obtain the short-time asymptotics for the solution of (1) which will be used to describe the source detection for very early arrivals to the cellular surface. This is a familiar problem from stochastic processes; the earliest arrivals are concentrated at the point closest to the source. Heuristically, this can be viewed as a boundary layer calculation. The outer solution is just the free space Green's function and the inner solution is confined to a boundary layer of width $\sqrt{Dt}$ at the edge of the sphere. Specifically, we will consider the problem when the diffusion length is much longer than the typical receptor size and spacing (so the homogenization approximation is valid) but much smaller that the sphere radius. We expect that the arrivals will be concentrated near the point on the sphere closest to the source, here taken as the polar axis (where $\theta = 0$).

The homogenized problem derived above allows a straightforward characterization of the fluxes at short times via the method of moments. Our starting point is the expansion of the Laplace transform of the flux density (5) as a Legendre series (reflecting the axisymmetry of the distribution). From the orthogonality of the Legendre modes,

$$\psi_n(c) = 2\pi \int_{\theta=0}^{\pi} \widehat{\mathcal{J}}(\theta, s) P_n(\cos\theta) \sin\theta\, d\theta, \qquad (8)$$

where $c = \sqrt{s/D}$. Next we exploit the exponential localization of the distribution to treat the interval $\theta \in [0, \pi]$ as effectively infinite. We define the moments of a radially symmetric two-dimensional distribution, $\mathcal{J}(\theta, t)$ as $M_n(t)$ and their Laplace transform $\widehat{M}_n(s) = \mathcal{L}[M_n(t)]$,

$$M_n(t) = 2\pi \int_{\theta=0}^{\pi} \mathcal{J}(\theta, t)\, \theta^n \, \sin\theta d\theta, \qquad \widehat{M}_n(s) = 2\pi \int_{\theta=0}^{\pi} \widehat{\mathcal{J}}(\theta, s)\, \theta^n \, \sin\theta d\theta. \tag{9}$$

The linearity of the moments implies that the Laplace transform of the moments are the moments of the Laplace transform. The axisymmetry implies the mean and the skewness of the distribution vanish and that the radial moments of interest are even n.

The zeroth moment is exactly $\psi_0(s)$

$$\widehat{M}_0(s) = \psi_0(c) = \frac{k_0(cR)/k_0'(c)}{k_0(c)/k_0'(c) - cD/\kappa} = \frac{e^{-(R-1)\sqrt{s/D}}}{R[1 + (D/\kappa)\cdot(1 + \sqrt{s/D})]},$$

whose inverse transform we have computed above

$$M_0(t) = \frac{\kappa}{R} e^{-\frac{(R-1)^2}{4Dt}} \left[\frac{1}{\sqrt{\pi D t}} - \mathrm{erfc}(\beta)\, e^{\beta^2} \left(\kappa/D + 1\right) \right], \tag{10}$$

where $\beta = \frac{R-1}{2\sqrt{Dt}} + (\kappa/D + 1)\sqrt{Dt}$. Assuming $t \ll 1$ which implies $\beta \gg 1$ and allowing that κ/D may be large yields the uniform approximation that

$$M_0(t) = \frac{\kappa}{R} \frac{e^{-\frac{(R-1)^2}{4Dt}}}{\sqrt{\pi D t}} \left[\frac{R-1}{(R-1) + 2\kappa t} \right] [1 + O(Dt)] \,.$$

To compute the higher moments, we make the ansatz that $\widehat{M}_{2k}(s) \sim \widehat{M}_0(s) c^{-2k}$. Expanding the first few Legendre polynomials near $\theta = 0$ yields

$$P_0(\cos\theta) = 1, \quad P_1(\cos\theta) = \cos\theta = 1 - \frac{1}{2}\theta^2 + \frac{1}{24}\theta^4 + \cdots, \quad P_2(\cos\theta) = \frac{3}{2}\cos^2\theta - \frac{1}{2} = 1 - \frac{3}{2}\theta^2 + \frac{1}{2}\theta^4 + \cdots$$

which yields

$$\widehat{M}_2(c) \sim -2\left[\chi_1(c) - \chi_0(c)\right] \sim 2\frac{R-1}{R^2} \frac{e^{-(R-1)\sqrt{s/D}}}{\sqrt{s/D}[1 + (D/\kappa)\sqrt{s/D})]} \cdot \left[1 + O\left(\frac{1}{\sqrt{s/D})} \right) \right],$$

whose inverse transform can be approximated for $\alpha, \beta \gg 1$ as

$$M_2(t) = M_0(t) \left[\frac{4Dt}{R} \right] \cdot \left[1 + O(\sqrt{Dt}) \right].$$

This allows us to compute the variance

$$\mathrm{Var}[\mathcal{J}(\theta, t)] \equiv \frac{M_2(t)}{M_0(t)} \sim \frac{4Dt}{R}.$$

A similar tedious calculation yields the result that

$$M_4(t) = M_0(t) \left[\frac{32(Dt)^2}{R^2} \right] \cdot \left[1 + O(\sqrt{Dt}) \right],$$

and shows that the kurtosis satisfies

$$\mathrm{Kur}[\mathcal{J}(\theta, t)] \equiv \frac{M_4(t)\cdot M_0(t)}{[M_2(t)]^2} \sim 2 + O(\sqrt{Dt}).$$

This is consistent with a Gaussian distribution, specifically, we have in the limit $t \to 0^+$ that

$$\mathcal{J}(\theta, t) \sim \frac{\mathcal{M}}{2\pi\sigma^2} e^{-\frac{\theta^2}{2\sigma^2}}; \qquad \sigma^2 = \frac{1}{2}\mathrm{Var}[\mathcal{J}(\theta, t)] \sim \frac{2Dt}{R}; \tag{11a}$$

$$\mathcal{M} = M_0(t) = \frac{\kappa}{R} \frac{\mathrm{e}^{-\frac{(R-1)^2}{4Dt}}}{\sqrt{\pi D t}} \left[\frac{R-1}{(R-1) + 2\kappa t} \right] [1 + O(Dt)] \,. \tag{11b}$$

Finally, if we are measuring a density, $J(\theta, t)$ that is solely a measure of the polar angle we need to integrate out the azimuthal component of the area element $dA = 2\pi \sin\theta \, d\theta$ which yields

$$J(\theta,t) = 2\pi \sin\theta \cdot \mathcal{J}(\theta,t) \sim 2\pi\theta\mathcal{J}(\theta,t) = \mathcal{M}\frac{\theta}{\sigma^2}e^{-\frac{\theta^2}{2\sigma^2}}.$$

In terms of the elevation coordinate $z = \cos\theta$, the transformed density is

$$J(z,t) = \frac{J(\theta,t)}{|dz/d\theta|} = \frac{\mathcal{M}}{\sigma^2}e^{-\frac{1-z}{\sigma^2}},$$

where we have used $\frac{\theta^2}{2} = 1 - z + O(\theta^4)$ for $\theta \ll 1$.

1.2 Asymptotics of scaling and centering parameters.

Here we develop and asymptotic solution to the nonlinear equation

$$h(t)e^{-\frac{(R-1)^2}{4Dt}} = M^{-1}, \tag{12a}$$

$$h(t) = A\frac{t^{\frac{3}{2}}}{(1+Bt)}, \qquad A = \sqrt{\frac{D}{\pi}}\frac{4\kappa}{R(R-1)^2}, \qquad B = \frac{2\kappa}{R-1}, \tag{12b}$$

as $M \to \infty$. We introduce the following rescaling of (12)

$$z^{-1}e^{-z}(1+\alpha z^{-1})^{-\frac{2}{3}} = \varepsilon, \qquad z = \frac{(R-1)^2}{6Dt}; \tag{13a}$$

$$\varepsilon = \frac{6D}{(R-1)^2}(MA)^{-\frac{2}{3}} = \left[\sqrt{\frac{27\pi}{2}}\frac{RD}{M\kappa(R-1)}\right]^{\frac{2}{3}}, \qquad \alpha = \frac{(R-1)^2}{6D}B = \frac{\kappa(R-1)}{3D} \tag{13b}$$

and solve (13) in the limit $\varepsilon \to 0$. After taking logarithms of (13a), we obtain an equation of form $z = f(z)$ and define the iterative scheme

$$z_{n+1} = f(z_n), \qquad f(z) = -\log\varepsilon - \log z - \frac{2}{3}\log\left(1+\alpha z^{-1}\right). \tag{14a}$$

Taking $\nu = -\log\varepsilon$, the first three iterations as $\nu \to \infty$ are

$$z_1 = \nu \tag{14b}$$

$$z_2 = f(z_1) = \nu - \log\nu - \frac{2}{3}\log\left(1+\frac{\alpha}{\nu}\right) \sim \nu - \log\nu - \frac{2\alpha}{3\nu} + O(\nu^{-2}) \tag{14c}$$

$$\begin{aligned} z_3 = f(z_2) &= \nu - \log\left[\nu - \log\nu - \frac{2\alpha}{3\nu}\right] - \frac{2}{3}\log\left(1+\alpha\left[\nu - \log\nu - \frac{2\alpha}{3\nu}\right]^{-1}\right) \\ &\sim \nu - \log\nu + \frac{\log\nu}{\nu} - \frac{2\alpha}{3\nu} + O\left(\frac{\log\nu}{\nu^2}\right). \end{aligned} \tag{14d}$$

From (13b), we therefore have the rescaled solution

$$t = \frac{(R-1)^2}{6Dz} = \frac{(R-1)^2}{6D\nu}\left[1 - \frac{\log\nu}{\nu} + \frac{\log\nu}{\nu^2} - \frac{2\alpha}{3\nu^2} + O\left(\frac{\log\nu}{\nu^3}\right)\right]^{-1}. \tag{15}$$

1.3 Optimal number of binding events.

In this section we explore the possibility for an optimizing number of binding events. We concentrate on characterizing the average elevation of the first K arrivals given by the variable $Z_K = \frac{1}{K}\sum_{i=1}^{K} z_i$. This variable, arising from a sum of exponential variables, follows a hypoexponential distribution which allows for a succinct analysis of its properties.

The value $Z_K = 1$ is the true source and we determine that the mean error satisfies

$$\mathbb{E}[1 - Z_K] = \sum_{i=1}^{K} \frac{\lambda_i}{K} = \frac{2D}{R}\Big(b_M + \frac{a_M}{K}\log K!\Big),$$

while the variance in the error is given by

$$\mathrm{Var}[1 - Z_K] = \sum_{i=1}^{K} \frac{\lambda_i^2}{K^2} = \frac{4D^2}{R^2K^2}\sum_{i=1}^{K}\Big(b_M + a_M \log i\Big)^2.$$

The errors increase in mean and decrease in variance as K increases. Can a balance of these trade-offs results in an optimal K? To explore this, we defined confidence intervals U_c for the hitting region $Z_K \in (z_c(K), 1)$ such that $\mathbb{P}[z_c(K) < Z_K < 1] = U_c$. Here the CDF $F_{Z_K}(z) = \mathbb{P}[z < Z_K < 1]$ is given by

$$F_{Z_K}(z) = \int_z^1 \mathbb{P}[Z_K = z']dz' = \sum_{i=1}^{K} w_i(1 - e^{-K(1-z)/\lambda_i}). \tag{16}$$

For several values of $U_c \in (0, 1)$, we obtained curves of $z_c(K)$ against K which are relatively flat but yield an optimizing value (Fig. 1). The flatness in of $z_c(K)$ is due to the slight decrease in $\mathbb{E}[1 - Z_K]$ as K increases, meaning that the extent of the hitting region is relatively insensitive to K. We observe the existence of an optimizing K only for $U_c \gtrapprox 0.77$. For such values of U_c where optimizing values of K exist, we find that these critical points depend quite sensitively on K. However, we do observe that only around $K \approx 5$ binding events are necessary to bring $z_c(K)$ quite close to the optimal value.

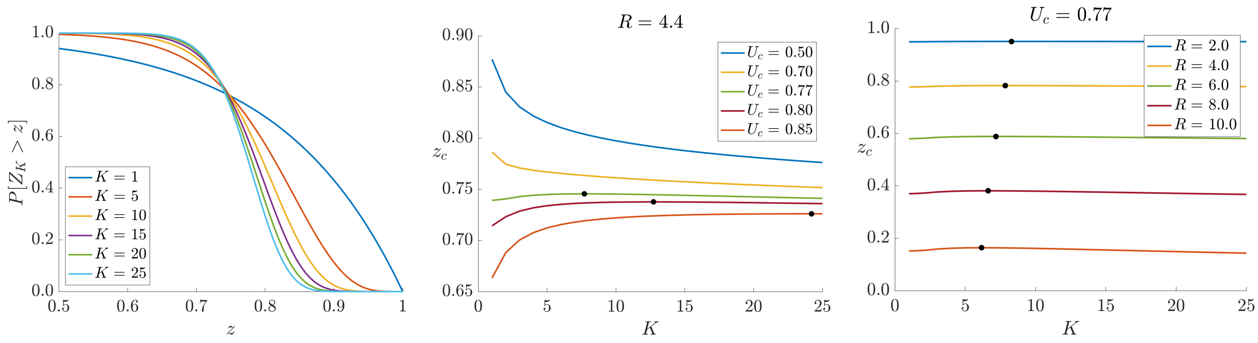

(a) $F_{Z_K}(z)$ for various K. (b) $z_c(K)$ for various U_c. (c) $z_c(K)$ for various R.

Figure 1: Exploration of optimizing number of binding events K. Left: The CDF $F_{Z_K}(z)$ for various increasing values of K. Center: Left boundary $z_c(K)$ of the hitting region $Z_K \in (z_c(K), 1)$ for various confidence intervals U_c and fixed value $R = 4.4$. Right: Plots of $z_c(K)$ for various R and fixed confidence interval $U_c = 0.77$.
