# Supplementary material for: Single-cell directional sensing from just a few receptor binding events
Source: Biophys J. 2023 Jun 24;122(15):3108–16. doi: 10.1016/j.bpj.2023.06.015 (PMC10432224; doi:10.1016/j.bpj.2023.06.015)
Supplement: Document S2. Article plus supporting material [file mmc2.pdf]

Biophysical Journal
Article

Biophysical Society

Single-cell directional sensing from just a few receptor binding events

Andrew J. Bernoff,[1] Alexandra Jilkine,[2] Adrián Navarro Hernández,[2] and Alan E. Lindsay[2,*]
[1]Department of Mathematics, Harvey Mudd College, Claremont, California and [2]Department of Applied & Computational Mathematics & Statistics, University of Notre Dame, South Bend, Indiana

ABSTRACT Identifying the directionality of signaling sources from noisy input to membrane receptors is an essential task performed by many cell types. A variety of models have been proposed to explain directional sensing in cells. However, many of these require significant computational and memory capacities for the cell. We propose and analyze a simple mechanism in which a cell adopts the direction associated with the first few membrane binding events. This model yields an accurate angular estimate to the source long before steady state is reached in biologically relevant scenarios. Our proposed mechanism allows for reliable estimates of the directionality of external signals using temporal information and assumes minimal computational capacities of the cell.

SIGNIFICANCE Directional sensing has been observed in many cell types, often at very low concentrations of chemical cues. Cells infer the direction of signaling sources through binding events at membrane bound surface receptors. Since only a few binding events to receptors can trigger a signaling cascade within the cell, we focus on early arrivals at receptor sites. We show that cells can acquire directional information before a steady state of the external chemical signal is reached. We propose a simple mechanism where a cell adopts the direction associated with the first few membrane binding events. This pre-steady-state response is in line with biological observations of cells responding to chemoattractants, or growth of fungi in response to extracellular signals.

INTRODUCTION

Accurately choosing a direction in which to move or grow in response to an external signal is an essential function of a variety of cell types. Examples of such behavior include chemotaxis (cell movement up a chemical gradient) (1–3), gradient-directed neuronal cone growth (4), and chemotropism (directed growth toward a pheromone source) (5–7). The determination of suitable direction in all these cases must be made from the noisy observations of binding of diffusing external signaling molecules to membrane bound receptors, coupled together with downstream intracellular amplification of the signal (1,7).

Since Berg and Purcell's pioneering paper on the physics of chemoreception (8), cellular sensitivity to external cues has long been considered through the paradigm of steady-state chemical gradients (9–12). As the limits of experimental measurements have expanded, effective directional sensing has been observed at lower and lower concentrations. Segall (5) first observed accurate orientation of yeast cells with concentrations of the pheromone α-factor of 67 nM. It was later demonstrated (13) that optimal directional sensing in yeast actually occurs at much lower 5 nM concentrations. Recent experimental studies (14) measured endogenous G-protein-coupled receptor activity in various cell types with high spatial and temporal resolution and established that GPC receptors are capable of responding to femtomolar and attomolar concentrations (10^{-15}–10^{-18} M). At such low concentrations, the cell must make decisions from just one or two receptor binding events (14). However, many theoretical models explaining cellular response to chemical cues are based on continuous representations of steady-state concentrations of chemoattractants.

In the context of directional sensing, recovering the source of external stimuli from steady-state receptor fluxes (so-called *splitting probabilities* (15)) is theoretically possible (16,17). Maximum likelihood estimation is a

Submitted April 11, 2023, and accepted for publication June 20, 2023.
*Correspondence: a.lindsay@nd.edu
Editor: Alex Mogilner.
https://doi.org/10.1016/j.bpj.2023.06.015
© 2023 Biophysical Society.
This is an open access article under the CC BY license (http://creativecommons.org/licenses/by/4.0/).

practical method to reconstruct source location from both steady-state (18) and dynamic receptor activity (19). Mechanisms for cellular implementation of maximum likelihood estimation in chemosensing have been proposed (20,21), suggesting biological feasibility; however, such an approach places significant computational requirements on a cell. The cell must know its geometry and the spatial configuration of its receptors, and it must store and integrate a temporal record of binding events. Can a simple mechanism assuming minimal cellular computational capacity provide an accurate directional estimate?

Here, we argue that a cell can reliably acquire accurate directional estimates of signaling sources by considering the earliest receptor binding events. These early events arise from diffusing signaling molecules that closely follow a straight line (shortest) trajectory from the source $\mathbf{x}_0$ to a receptor (22,23), and hence these paths convey significant directional information to the cell. From a combination of homogenization theory, extreme value theory, and short-time asymptotics for the diffusion equation, we show that an estimate based on the position of the first binding event is highly accurate, provided the source is not too distant. If instead one considers the distribution of the first few arrivals, we find that, while the mean error slowly increases, the variance is significantly reduced.

Model

We consider the simple conceptual model of Berg-Purcell (8), which continues to serve as a bedrock for understanding receptor activation (24–27). Let $\Omega \subset \mathbb{R}^3$ be the unit sphere with N static, circular, nonoverlapping surface receptors of common radius a (cf. Fig. 1). The organization of surface receptors can vary from spatially homogeneous (e.g., GABA (28)) to clustered (e.g., yeast (29)). Clustered (18) and diffusing (30) receptor configurations can be incorporated within this framework; however, we consider here a fixed and spatially uniform configuration of receptors reflective of a cell in a quiescent state. The receptors occupy a surface fraction $\sigma = Na^2/4$ and their fixed locations are centered at the Fibonacci spiral points, a well-known and effective covering of the sphere (31,32). To explore the efficacy of using early binding events to infer source direction, we consider M diffusing particles originating at $\mathbf{x}_0 = (0,0,R)$ for $R > 1$. A number (M_a) of these particles will reach and bind to a receptor while the remaining $(M - M_a)$ will escape to infinity. We simulate this process on a triangulated cell surface and use a particle-based diffusion simulation with kinetic Monte Carlo acceleration (33,34). The arrival times $\{t_k\}_{k=1}^{M_a}$ are sorted $(t_k < t_{k+1})$ and the associated binding locations $\{\mathbf{x}_k\}_{k=1}^{M_a}$ recorded. The source is located on the polar axis, with associated unit vector $\mathbf{e}_3 = (0,0,1)$, and we characterize events via the elevation $z_k = \cos\theta_k = \mathbf{e}_3 \cdot \mathbf{x}_k$, where θ_k is the angle between the North Pole and the location of the kth binding event. Values $z_k = \cos\theta_k \sim 1$ indicate alignment with the source, while $z_k \sim 0$ indicates a uniform distribution over the sphere. Using $M = 10^5$ initial particles for $R = 5$, we calculate points $(\bar{t}_n, \bar{z}_n)$ consisting of a running average of M_s elevations, specifically

$$\bar{t}_n = \frac{1}{M_s}\sum_{k=n}^{n+M_s-1} t_k, \quad \bar{z}_n = \frac{1}{M_s}\sum_{k=n}^{n+M_s-1} \cos\theta_k. \quad (1)$$

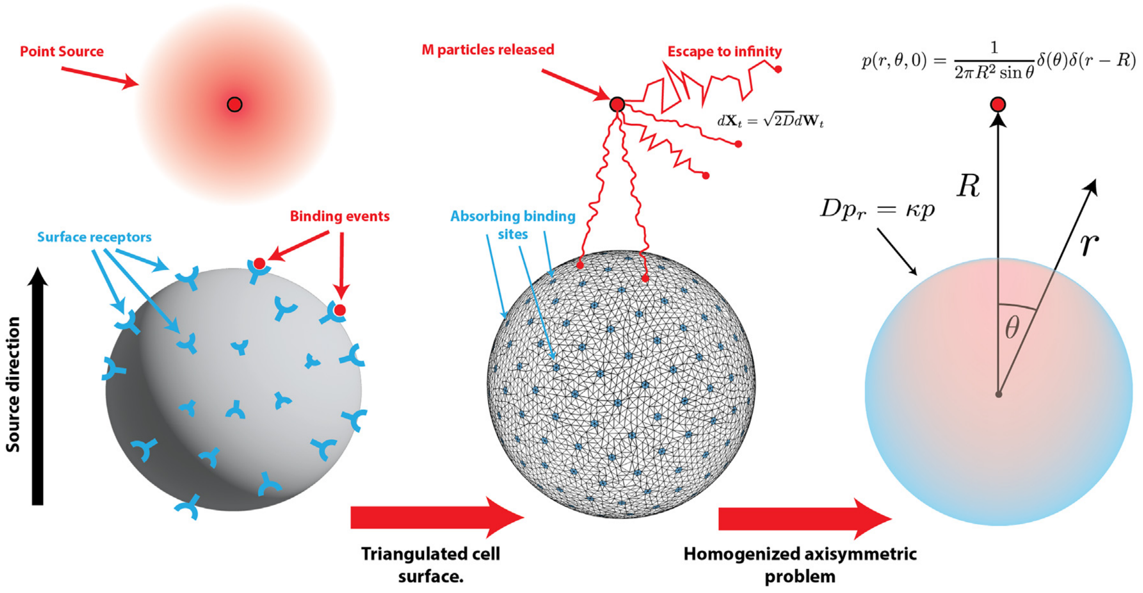

FIGURE 1 Model. Left: binding events at cell surface receptors give information on source direction. Center: our kinetic Monte Carlo model uses a triangulated cell surface with M diffusing particles released at $t = 0$ from the source at $\mathbf{x}_0 = (0,0,R)$. Binding events occur when particles reach small circular absorbing sites on the cell surface. Right: axisymmetric continuous formulation as a diffusion equation with a Dirac source at $(0,0,R)$ and homogenized boundary condition $Dp_r = \kappa p$ on the cell boundary $r = 1$. To see this figure in color, go online.

In Fig. 2, we plot Eq. 1 for values $M_s = \{11, 101, 1001\}$. At short times we observe that elevations $\bar{z} \sim 1$, corresponding to binding events aligned in the source direction. At later times, particles gradually lose information on their initial position and binding events occur uniformly on the surface ($\bar{z} \sim 0$ as $t \to \infty$). Increasing the number of averaging points M_s reduces the variance. This simulation suggests that, at short times, the directions associated with binding events give an estimate strongly biased toward the source. How short is "short enough" and how accurate is such an estimate? To answer this, we analyze a homogenized partial differential equation (PDE) model.

Homogenization

Boundary homogenization theory (19,34–39) posits that the complex configuration of surface receptors and associated mixed boundary conditions can be replaced by the Robin condition, $D\partial_\nu p = \kappa p$ on $\partial\Omega$, where $\partial_\nu \equiv \hat{\mathbf{n}} \cdot \nabla$ is the normal derivative. For $\mathbf{x}_0 = (0, 0, R)$, the axisymmetric particle density $p(r, \theta, t; R)$ solves the initial boundary value problem

$$\frac{\partial p}{\partial t} = D\left[\frac{\partial^2 p}{\partial r^2} + \frac{2}{r}\frac{\partial p}{\partial r} + \frac{1}{r^2 \sin\theta}\frac{\partial^2 p}{\partial \theta^2}\right], \; t > 0, \; r > 1, \; 0 < \theta < \pi; \tag{2a}$$

$$D\partial_r p = \kappa p, \; t > 0, \; r = 1, \; 0 < \theta < \pi; \tag{2b}$$

$$p = \frac{1}{2\pi R^2 \sin\theta}\delta(r - R)\delta(\theta), \; t = 0, \; r > 1, \; 0 < \theta < \pi \tag{2c}$$

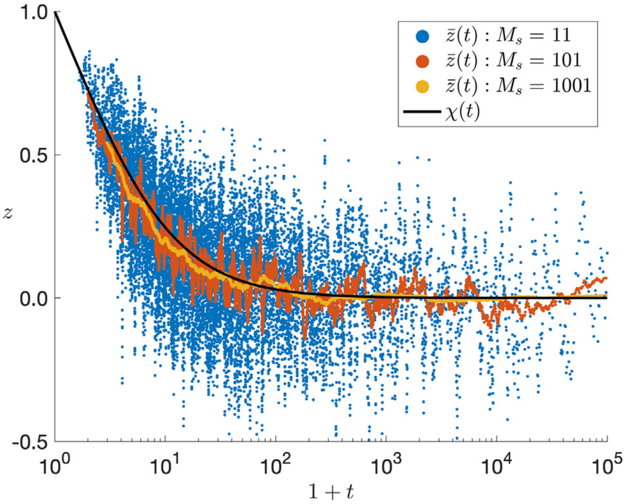

FIGURE 2 The average elevation coordinate (Eq. 1) of binding events on the membrane for $R = 5, N = 201, D = 1, \sigma = 0.05$, and $M = 10^5$. Variance reduction is achieved by increasing the number of binding events, M_s in the running average. The parameter from Eq. 3 is $\kappa \approx 3.36$, which yields the homogenized theoretical prediction $\chi(t)$ from Eq. 7. To see this figure in color, go online.

In our recent work (19), we considered a circular two-dimensional cell and established that the correct homogenization of the full time-dependent dynamics is given by the established homogenization of the steady-state problem (40,41). Here, we conjecture and numerically verify in Fig. 3 that a similar result holds for a spherical cell. Specifically, for the case of uniformly distributed receptors with combined surface fraction $\sigma \ll 1$, we posit that the density can be recovered by applying the Robin condition (Eq. 2b) with

$$\kappa = \frac{4\sigma}{\pi a}\left[1 - \frac{4}{\pi}\sqrt{\sigma} + \frac{a}{\pi}\log\left(4\sqrt{\sigma}\, e^{-\frac{1}{2}}\right) + \frac{a^2}{2\pi\sqrt{\sigma}}\right]^{-1}, \tag{3}$$

where Eq. 3 is the homogenization parameter derived from the steady-state flux (38,42,43). The solution of Eq. 2 is separable and available in terms of spherical Bessel expansions. We find (cf. supporting material) the surface flux $\mathcal{J} = D\partial_r p|_{r=1}$ to be

$$\mathcal{J}(\theta, t) = \frac{1}{2\pi}\sum_{n=0}^{\infty}\frac{2n+1}{2}\psi_n(t; R)P_n(\cos\theta). \tag{4a}$$

Here, $P_n(z)$ are the Legendre polynomials. The coefficients are determined through Laplace transform $\hat{\psi}_n(s; R) = \int_{t=0}^{\infty}\psi_n(t; R)e^{-st}dt$ and are given by

$$\hat{\psi}_n(s; R) = \frac{k_n(cR)}{k_n(c) - c\frac{D}{\kappa}k_n'(c)}, \quad c = \sqrt{\frac{s}{D}}. \tag{4b}$$

Here, $k_n(z)$ is the modified spherical Bessel function of the second kind. The total flux $\rho(t) = 2\pi\int_{\theta=0}^{\pi}\mathcal{J}(\theta, t)\sin\theta d\theta$ is

$$\rho(t) = \frac{\kappa}{R}e^{\frac{-(R-1)^2}{4Dt}}\left[\frac{1}{\sqrt{\pi D t}} - \left(\frac{\kappa}{D} + 1\right)\mathrm{erfcx}(\beta)\right], \tag{5}$$

where $\beta = (R-1)/\sqrt{4Dt} + \left(\frac{\kappa}{D} + 1\right)\sqrt{Dt}$ and $\mathrm{erfcx}(z) = \frac{2}{\sqrt{\pi}}e^{z^2}\int_z^{\infty}e^{-t^2}\;dt$ is the scaled complementary error function. We remark that

$$\int_0^{\infty}\rho(t)dt = \frac{1}{\left(1 + \frac{D}{\kappa}\right)R}, \tag{6}$$

so that the probability of capture is not unity, but inversely proportional to the initial distance to the sphere. In fact, this result provides the expected fraction of binding events $\mathbb{E}[M_a] = M/[(1 + D/\kappa)R]$. In Fig. 3, we demonstrate with comparison to Monte Carlo simulations that the homogenized solution accurately predicts the capture rate. At what time do binding events cease to convey information on the source direction? The answer can be gleaned by considering from Eq. 4a the average elevation of events at time t given by

$$\chi(t) = \frac{\int_{\theta=0}^{\pi}\cos\theta\,\mathcal{J}(\theta, t)\sin\theta\, d\theta}{\int_{\theta=0}^{\pi}\mathcal{J}(\theta, t)\sin\theta\, d\theta} = \frac{\psi_1(t)}{\psi_0(t)}. \tag{7}$$

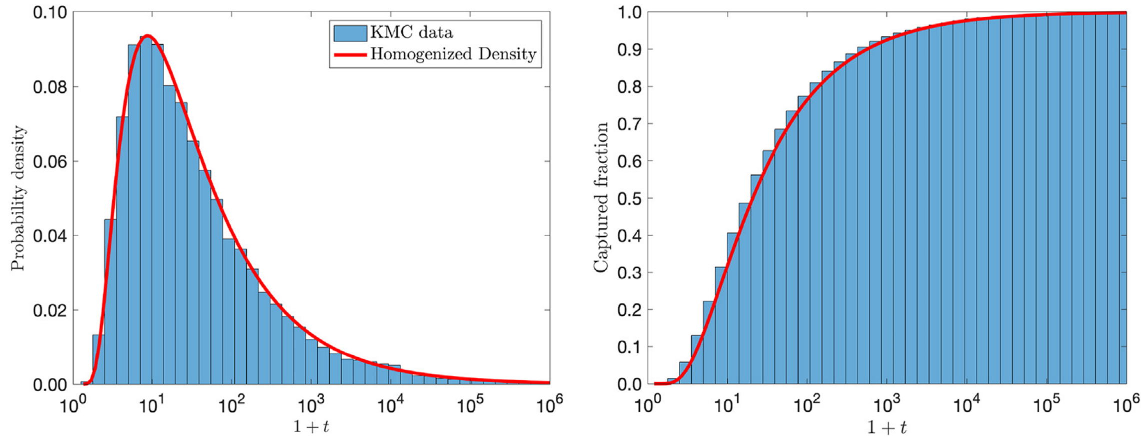

FIGURE 3 Validation of boundary homogenization for $R = 5, N = 201, \sigma = 0.05, D = 1$, and $M = 10^5$. The formula Eq. 3 gives $\kappa \approx 3.36$. The full distribution of arrival times (*left*) and the cumulative density function of captured particles (*right*) from Monte Carlo (*histograms*) and homogenization theory Eq. 5 (*solid red*). To see this figure in color, go online.

At large times the function $\chi(t)$ tends to zero as the surface flux becomes radially symmetric and consequently arriving particles yield no information on the source direction. In Fig. 2 we see that $\chi(t)$ agrees well with numerical results and thus predicts the time-dependent bias of the surface flux toward the source. The close agreement in Fig. 2 provides an additional nontrivial validation of the boundary homogenization approach.

Extreme arrivals

We have observed that averaging of binding events at short times gives an estimate biased toward the source. We now focus on calculating the distribution of the times and locations of the earliest binding events, usually referred to as *extreme arrivals*. Our previous study (19) explored dynamics two-dimensionally with characterization of equilibrium dynamics and short-time fluxes. Here, we consider the distribution of the kth arrival to the sphere in three dimensions. Specifically, consider M particles released from $\mathbf{x}_0 = (0,0,R)$, which results in binding events at times $\{t_k\}_{k=1}^{M_a}$ with $t_k < t_{k+1}$. Lawley (23) determined for $M \gg 1$ that t_k follows the Gumbel distribution with mean and variance determined by the limiting behavior of the survival probability $S(t) = \mathbb{P}[t_1 < t]$ as $t \to 0^+$. We calculate (cf. supporting material) that $S(t) \sim 1 - h(t)\exp\left[-\frac{(R-1)^2}{4Dt}\right]$ as $t \to 0^+$ where

$$h(t) = \sqrt{\frac{D}{\pi}}\frac{4\kappa}{R(R-1)}\left[\frac{t^{\frac{3}{2}}}{(R-1)+2\kappa t}\right][1+\mathcal{O}(Dt)]. \quad (8)$$

Applying ((23), Prop 3, and Thm 4.), we have that

$$\frac{t_k - b_M}{a_M} \to X_k, \text{ as } M \to \infty, \quad (9)$$

where the density of X_k is given by

$$\mathbb{P}[X_k = x] = \frac{e^{kx - e^x}}{(k-1)!}. \quad (10)$$

The centering and scaling parameters, b_M and a_M, respectively, are given by the relations

$$S(b_M) = 1 - M^{-1}, \quad a_M = -(MS'(b_M))^{-1}. \quad (11)$$

Eq. 11 is amenable to numerical and asymptotic solution for $M \gg 1$ (cf. supporting material). At leading order

$$b_M \sim \frac{(R-1)^2}{6D\nu}, \quad \nu = \log\left[\sqrt{\frac{2}{27\pi}}\frac{M\kappa(R-1)}{RD}\right]^{\frac{2}{3}}. \quad (12)$$

We calculate the distribution of the angle θ_k to be

$$\mathbb{P}[\theta_k = \eta] = \int_{\tau=0}^{\infty}\mathbb{P}[\theta = \eta | t = \tau]\mathbb{P}[t_k = \tau]d\tau \approx \frac{\mathcal{J}[\theta = \eta | t = b_M + a_M \log k]}{\rho(b_M + a_M \log k)}. \quad (13)$$

The approximation leading to Eq. 13 is based on Laplace's method with evaluation at $\text{Mode}[X_k] = b_M + a_M \log k$, the value around which the distribution is highly peaked. In terms of $z = \cos\theta$, we determine (cf. supporting material) as $t \to 0^+$ that

$$\mathcal{J}(z,t) \sim \frac{\rho(t)}{\lambda}e^{-\frac{1-z}{\lambda}}, \quad \lambda = \frac{2Dt}{R}; \quad (14a)$$

$$\rho(t) \sim \frac{\kappa}{R}\frac{e^{-\frac{(R-1)^2}{4Dt}}}{\sqrt{\pi Dt}}\left[\frac{R-1}{(R-1)+2\kappa t}\right], \quad (14b)$$

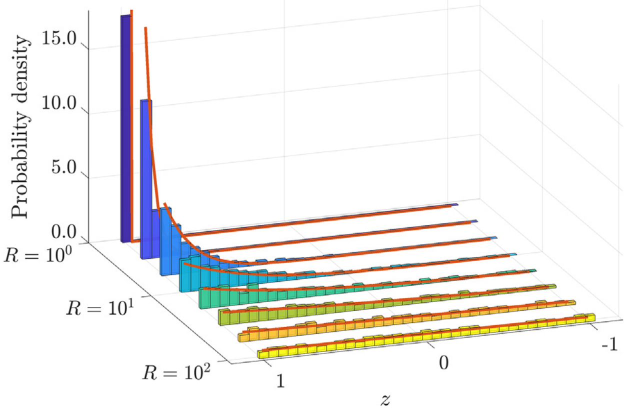

FIGURE 4 Distribution of locations for first binding event $z_1 = \cos\theta_1$ at eight equally log-spaced points from $R = 10^0$ to $R = 10^2$. Parameters $\kappa = 3.36$, $D = 1$, and $M = 10^5$. Histograms based on 1000 independent realizations with theoretical predictions (Eq. 4) in close agreement (*solid red*). To see this figure in color, go online.

Combining Eq. 13 and Eq. 14, we conclude for $M \gg 1$ that the elevation from the North Pole of the kth binding event has exponential distribution $(1 - z_k) \sim \text{Exp}(\lambda_k^{-1})$ with

$$\lambda_k = \frac{2D}{R}[b_M + a_M \log k]. \tag{15}$$

Smaller values of λ_k are associated with a more accurate directional estimate. Since $\lambda_k < \lambda_{k+1}$, the directional information of the first event $(k = 1)$ yields the lowest error and smallest variance compared with subsequent events $(k > 1)$. Further applying Eq. 12 in the limit as $M \to \infty$, we obtain

$$\lambda_1 \sim \frac{(R-1)^2}{3R\nu} \sim \frac{(R-1)^2}{2R \log M}. \tag{16}$$

The form of Eq. 16 reveals that lower errors are associated with large M and small values of $(R - 1)$. Hence, we conclude that a cell can make an accurate estimate of a source direction from just a single binding event, provided $(R-1)$ is not too large. In Fig. 4 we show numerical validation of the distribution of z_1 as predicted by Eq. 14 based on 1000 independent realizations and parameter values $\kappa = 3.34$, $D = 1$, and $M = 10^5$.

Multiple binding events

Could an improvement to this simple estimate be formed by considering several early binding events? The average elevation of the first K events is given by the variable $Z_K = \frac{1}{K}\sum_{i=1}^{K} z_i$. We determine that $1 - Z_K \sim \text{Hypo}\left(\frac{K}{\lambda_1}, \ldots, \frac{K}{\lambda_K}\right)$ where a *hypoexponential* variable $Z \sim \text{Hypo}(\alpha_1, \ldots, \alpha_K)$ has density

$$\mathbb{P}[Z = z] = \sum_{i=1}^{K} w_i \alpha_i e^{-\alpha_i z}, \quad w_i = \prod_{\substack{j=1 \\ j \neq i}}^{K} \frac{\alpha_j}{\alpha_j - \alpha_i}. \tag{17}$$

The mean and variance of the error $1 - Z_K$ are calculated to be

$$\mathbb{E}[1 - Z_K] = \sum_{i=1}^{K} \frac{\lambda_i}{K} = \frac{2D}{R}\left(b_M + \frac{a_M}{K}\log K!\right), \tag{18a}$$

$$\text{Var}[1 - Z_K] = \sum_{i=1}^{K} \frac{\lambda_i^2}{K^2} = \frac{4D^2}{R^2K^2}\sum_{i=1}^{K}(b_M + a_M \log i)^2. \tag{18b}$$

We hence conclude from Eq. 18 that taking the average of K early arrivals results in a slight precession of the mean away from the correct value $Z_K = 1$, but yields a smaller variance, therefore generating a tighter distribution. We demonstrate this effect in Fig. 5 for the parameters $R = 4.4$, $M = 10^5$, $D = 1$, $\kappa = 3.36$ with data shown for 1000 realizations. The distribution of arrivals from the first $(K = 1)$ arrival is exponential and the average of the first $K = 10$ arrivals is hypoexponential. As demonstrated in the inset of Fig. 5, an increase in the number of events K averaged yields a slight deterioration in the average error but a large reduction in the variance. For example, averaging over just $K = 5$ events increases the mean error by approximately 11% compared with the first arrival, but decreases the error variance by roughly a factor of 4. The trade-off between the error's increasing mean (Eq. 18a) and decreasing variance (Eq. 18b) as a function of K suggests that there may be an optimal number of binding events. In the supporting material we explore this trade-off.

Application to chemosensing

Budding yeast *Saccharomyces cerevisiae* is a well-studied model system for chemosensing. In *S. cerevisiae* polarized growth toward a mating partner is guided by diffusible chemoattractants, such as α-factor (7). Experimental studies have reported that yeast cells are capable of sensing gradients as shallow as 0.1 nM/μm (13,44) and do so over 1000-fold range of α-factor concentrations. Measured $\cos\theta$ values of yeast mating projections (13), analogous to our estimated chemoattractant coordinate (cf. Fig. 2), show gradient detection accuracy was maximized for $\cos\theta \approx 0.8$ in $0 - 5$ nM concentration gradient. Accuracy decreases at higher α-factor concentrations and shallower gradients, but the cells are able to sense direction over a broad range of concentrations and gradient slopes, even at concentrations as high as 1 μM, with the typical $\cos\theta \approx 0.6$, and in a uniform gradient $\cos\theta \approx 0$ as expected. It is important to note that microfluidic experiments done by

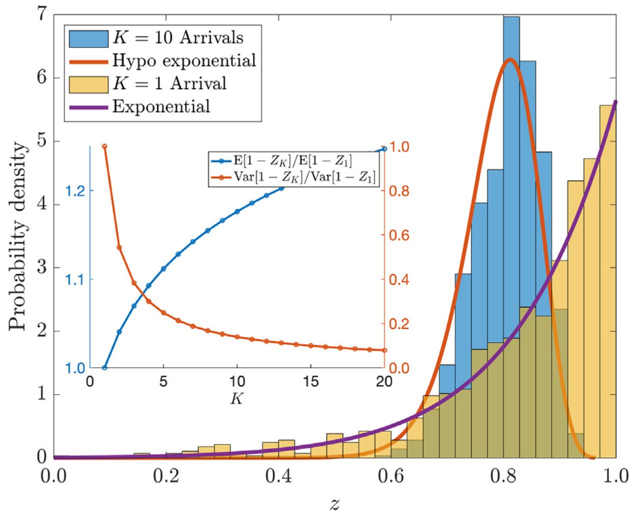

FIGURE 5 Demonstration of variance reduction from averaging several binding events with parameters $R = 4.4$, $\kappa = 3.36$, $D = 1$, and $M = 10^5$. Outer figure: distributions of Z_K for $K = 10$ and $K = 1$ based on 1000 realizations. Inset: relative changes in mean and variance of estimate for a range of K values. As K increase the mean error (Eq. 18a) increases slowly while the variance (Eq. 18b) can be reduced substantially. To see this figure in color, go online.

(13) used a steady-state gradient, while our model and simulations use a point source, and are a better representations of experiments by (5), who first demonstrated that yeast cells could sense a chemoattractant source emitted using a micropipette and form a projection toward the source.

The binding dynamics of α-factor to its receptor are known to be slow. At concentrations near the dissociation constant ($K_d \approx 5$ nM), binding takes about 20 min to reach 90% of the equilibrium level (45). However, the yeast cell starts responding to the chemoattractant by assembling a polarity site within minutes, and later potentially moves the polarity site to track the chemotactic source on a longer timescale. This supports our hypothesis that chemosensing starts long before a steady-state gradient may be reached. As noted by (45), extracting information pre-equilibrium can overcome both the noise issue and receptor saturation limits, and expand the input dynamic range of chemoattractant to which budding yeast cells can respond.

Another example of directional sensing occurs in chemotactic cells such as *Dictyostelium discoideum* and neutrophils, where, when a chemoattractant is released from a pipette, a response is observed in 2–5 s (1,46,47). Thus, the first step in chemosensing must occur on this timescale. Our predictions of extreme statistics are of roughly the same order of magnitude as those observed experimentally for *Dictyostelium* and provide a minimum time for a cell to respond to a diffusive signal.

Chemosensing involves the cell surface receptors binding to extracellular diffusing molecules. What is the typical receptor number N on different types of cells that need to respond to external chemical sources? Physiological receptor numbers vary considerably: $N \approx 10^2 - 10^3$ in receptors in neural cone growth (4,28), $N \approx 10^4$ in budding yeast (48), and $N \approx 10^4 - 10^5$ in lymphocytes (49). Activation of these receptors then induces the production of second-messenger molecules that then transduce the external signal to downstream signaling cascades within a cell. In our model, we considered chemotactic source molecules diffusing in a three-dimensional region and interacting with receptors uniformly embedded on the surface of a single spherical cell. We have not considered the effects of receptor clustering, extracellular ligand unbinding from the receptor once a binding event occurs, receptor internalization, and the duration of receptor being in a bound state to the ligand. All these effects are potentially important in different biological contexts (36,50). For example, ratiometric sensing, by considering the ratio of active to inactive receptors, compensates for uneven receptor density in *S. cerevisiae* and allows more accurate gradient detection (6,7). At steady state, the fraction of bound receptors depends on both the external chemical concentration and the binding dissociation constant K_d. Interestingly, (50) uses a particle-based reaction-diffusion model of *S. cerevisiae* ligand-receptor dynamics and reports that neither time-averaging nor receptor endocytosis significantly improves the cell's accuracy in detecting gradients over timescales associated with the initiation of polarized growth in yeast. Our proposed chemo-detection mechanism does not consider the putative positive feedback loops downstream of receptors that allow cells to fine tune its chemosensing machinery on a longer timescale (29,51,52). However, this means that there are no unknown parameters to fit in our phenomenological model, which is often not the case for more detailed mechanistic models.

DISCUSSION

In this paper we propose and analyze a simple method for directional sensing based on the estimates formed by the first few binding events of signaling molecules to membrane receptors. In contrast to limits imposed on gradient sensing at equilibrium, we shown here that, long before equilibrium is attained, these early receptor binding events confer sufficient information to accurately estimate the source direction. Among M events, we characterize the distribution associated with the average of the first K arrivals for $1 \leq K \ll M$ as hypoexponential. A directional estimate based on just the first binding is accurate, provided the source is not too distant. Since we focus on the initial few binding events, the issue of receptor saturation, which can occur at high chemoattractant concentrations and can be potentially alleviated by receptor recycling, is not an issue on the timescales that we are considering. From our analysis, we expect that signal strength (M) and source distance (R) are the main factors that determine the efficacy of extreme statistics in directional sensing. Factors, such as receptor binding/unbinding, can be thought of as modulating

κ, which our analysis shows to be a lower-order effect (36). Hence, when one is interested only in the distribution of the first few binding events, mean receptor occupancy by the ligand does not play a large role.

Gradient sensing strategies fall into two major categories: temporal and spatial. Temporal sensing mechanisms, thought to be used by bacteria, involve an organism moving and sampling the concentration of chemoattractant in its environment. Spatial sensing mechanisms, in which the organism compares receptor occupancy difference across the cell body, are thought to be used by larger eukaryotic chemotactic cells (1,9). The fact that yeast cells are not motile has been used to suggest that they also use a spatial sensing mechanism, despite being smaller (4 μm in diameter) than most eukaryotic cells (7). However, as we show here, an immobile cell can still use temporal information to help it detect a chemotactic source before a steady state is reached. Our model has some similarities to the "first hit" model proposed by (53). In that work, initial activation of receptors activates the side of the cell closest to the stimulus and triggers a rapid inhibitory response that spreads across the cell and prevents the posterior from responding. When the gradient is repositioned, there is again an initial contact and the direction of the response is reset. Most modeling literature on chemosensing in eukaryotes uses deterministic models and assumes that the signal from the chemotactic source is at equilibrium (54). Here, we argue that immobile cells can actually acquire a lot of information from the time-dependent problem. Chemotactic cells can orient toward a micropipette source on a very rapid timescale, and this process also occurs in immobilized cells that cannot undergo cell shape change (1). Cellular response toward the source of chemical cues has been also been observed only a few seconds after exposure to a chemoattractant (47). It would be interesting to combine the stochastic direction-sensing mechanism we propose here to some of the proposed models for gradient amplification and cell polarization downstream of the receptors (11,54–57) and predict the frequency of reorientation of the cell to a changing source position.

Sensing of multiple sources with general spatial and temporal distributions is a natural extension of this work. The linearity of the underlying problem allows for superposition of the solutions developed here. In such a scenario, the surface flux $\mathcal{J}(\theta, t)$ would exhibit multiple peaks in space and time whose structure would need to be resolved. Similar recovery analysis has been accomplished using Fourier methods in the context of defect localization (58). In addition, it would also be interesting to consider the effect of nonspherical cell geometry on chemosensing for cell types such as *Dictyostelium* and neutrophils.

Finally, directional sensing is often performed in a group of cells, with putative feedback from other cells (59–63). Another important biological problem, where multiple cells need to determine their position within a tissue due to an external chemical gradient of a *morphogen* (64), has traditionally assumed due to the timescale of development that the morphogen gradient is at steady state (65,66). However, there is increasing biological evidence that some morphogen gradients may actually start being interpreted before reaching steady state (67–69). Theoretical work suggests that pre-steady-state measurements of the morphogen gradient may reduce the effects of stochastic fluctuations on determining spatial boundaries in tissue (70). It would be interesting to revisit our proposed chemosensing mechanism for a group of cells.

SUPPORTING MATERIAL

Supporting material can be found online at https://doi.org/10.1016/j.bpj.2023.06.015.

AUTHOR CONTRIBUTIONS

A.E.L. and A.B. designed the research, performed research, and wrote the paper. A.J. analyzed the data and wrote the paper. A.N.H. analyzed the data and performed the research.

ACKNOWLEDGMENTS

A.E.L. is supported by NSF DMS-1815216. A.J. is supported by NSF DMS-2052687. We thank the anonymous referees for their careful reading of the manuscript and helpful suggestions.

DECLARATION OF INTERESTS

The authors declare no competing interests.

REFERENCES

1. Parent, C. A., and P. N. Devreotes. 1999. A cell's sense of direction. *Science.* 284:765–770.
2. Song, L., S. M. Nadkarni, …, E. Bodenschatz. 2006. Dictyostelium discoideum chemotaxis: Threshold for directed motion. *Eur. J. Cell Biol.* 85:981–989.
3. Varennes, J., H.-r. Moon, …, B. Han. 2019. Physical constraints on accuracy and persistence during breast cancer cell chemotaxis. *PLoS Comput. Biol.* 15:e1006961.
4. Goodhill, G. J. 2016. Can Molecular Gradients Wire the Brain? *Trends Neurosci.* 39:202–211. https://doi.org/10.1016/j.tins.2016.01.009.
5. Segall, J. E. 1993. Polarization of yeast cells in spatial gradients of alpha mating factor. *Proc. Natl. Acad. Sci. USA.* 90:8332–8336.
6. Henderson, N. T., M. Pablo, …, D. J. Lew. 2019. Ratiometric GPCR signaling enables directional sensing in yeast. *PLoS Biol.* 17:e3000484.
7. Ghose, D., T. Elston, and D. Lew. 2022. Orientation of cell polarity by chemical gradients. *Annu. Rev. Biophys.* 51:431–451.
8. Berg, H. C., and E. M. Purcell. 1977. Physics of chemoreception. *Biophys. J.* 20:193–219.
9. Levchenko, A., and P. A. Iglesias. 2002. Models of eukaryotic gradient sensing: Application to chemotaxis of amoebae and neutrophils. *Biophys. J.* 82:50–63. https://doi.org/10.1016/S0006-3495(02)75373-3.

10. Endres, R. G., and N. S. Wingreen. 2008. Accuracy of direct gradient sensing by single cells. *Proc. Natl. Acad. Sci. USA*. 105:15749–15754. https://doi.org/10.1073/pnas.0804688105.

11. Levine, H., D. A. Kessler, and W.-J. Rappel. 2006. Directional sensing in eukaryotic chemotaxis: a balanced inactivation model. *Proc. Natl. Acad. Sci. USA*. 103:9761–9766.

12. Rappel, W. J., and H. Levine. 2008. Receptor noise and directional sensing in eukaryotic chemotaxis. *Phys. Rev. Lett.* 100:228101–228109.

13. Moore, T. I., C.-S. Chou, ..., T.-M. Yi. 2008. Robust spatial sensing of mating pheromone gradients by yeast cells. *PLoS One*. 3, e3865.

14. Civciristov, S., A. M. Ellisdon, ..., M. L. Halls. 2018. Preassembled GPCR signaling complexes mediate distinct cellular responses to ultra-low ligand concentrations. *Sci. Signal.* 11, eaan1188.

15. Metzler, R., G. Oshanin, and S. Redner. 2014. First-Passage Phenomena and Their Applications. World Scientific.

16. Dobramysl, U., and D. Holcman. 2021. Reconstructing a point source from diffusion fluxes to narrow windows in three dimensions. *Proc. R. Soc. A*. 477, 20210271.

17. Dobramysl, U., and D. Holcman. 2018. Mixed analytical-stochastic simulation method for the recovery of a Brownian gradient source from probability fluxes to small windows. *J. Comput. Phys.* 355:22–36. http://www.sciencedirect.com/science/article/pii/S0021999117308252.

18. Lawley, S. D., A. E. Lindsay, and C. E. Miles. 2020. Receptor Organization Determines the Limits of Single-Cell Source Location Detection. *Phys. Rev. Lett.* 125, 018102. https://doi.org/10.1103/PhysRevLett.125.018102.

19. Lindsay, A. E., A. J. Bernoff, and A. Navarro Hernández. 2023. Short-time diffusive fluxes over membrane receptors yields the direction of a signalling source. *R. Soc. Open Sci.* 10, 221619.

20. Endres, R. G., and N. S. Wingreen. 2009. Maximum Likelihood and the Single Receptor. *Phys. Rev. Lett.* 103, 158101. https://doi.org/10.1103/PhysRevLett.103.158101.

21. Aquino, G., N. S. Wingreen, and R. G. Endres. 2016. Know the Single-Receptor Sensing Limit? Think Again. *J. Stat. Phys.* 162:1353–1364. http://link.springer.com/10.1007/s10955-015-1412-9.

22. Lawley, S. D., and J. B. Madrid. 2020. A Probabilistic Approach to Extreme Statistics of Brownian Escape Times in Dimensions 1, 2, and 3. *J. Nonlinear Sci.* 30:1207–1227. https://doi.org/10.1007/s00332-019-09605-9.

23. Lawley, S. D. 2020. Distribution of extreme first passage times of diffusion. *J. Math. Biol.* 80:2301–2325. https://doi.org/10.1007/s00285-020-01496-9.

24. Shoup, D., and A. Szabo. 1982. Role of diffusion in ligand binding to macromolecules and cell-bound receptors. *Biophys. J.* 40:33–39.

25. Endres, R. G., and N. S. Wingreen. 2009. Accuracy of direct gradient sensing by cell-surface receptors. *Prog. Biophys. Mol. Biol.* 100:33–39. https://doi.org/10.1016/j.pbiomolbio.2009.06.002.

26. Northrup, S. H. 1988. Diffusion-controlled ligand binding to multiple competing cell-bound receptors. *J. Phys. Chem.* 92:5847–5850. https://doi.org/10.1021/j100331a060.

27. Bressloff, P. C., and J. M. Newby. 2013. Stochastic models of intracellular transport. *Rev. Mod. Phys.* 85:135–196. https://doi.org/10.1103/RevModPhys.85.135.

28. Bouzigues, C., M. Morel, ..., M. Dahan. 2007. Asymmetric redistribution of GABA receptors during GABA gradient sensing by nerve growth cones analyzed by single quantum dot imaging. *Proc. Natl. Acad. Sci. USA*. 104:11251–11256.

29. Ismael, A., W. Tian, ..., D. E. Stone. 2016. G βpromotes pheromone receptor polarization and yeast chemotropism by inhibiting receptor phosphorylation. *Sci. Signal.* 9:1–17.

30. Lawley, S. D., and C. E. Miles. 2019. How Receptor Surface Diffusion and Cell Rotation Increase Association Rates. *SIAM J. Appl. Math.* 79:1124–1146.

31. González, Á. 2009. Measurement of Areas on a Sphere Using Fibonacci and Latitude–Longitude Lattices. *Math. Geosci.* 42:49–64. https://doi.org/10.1007/s11004-009-9257-x.

32. Swinbank, R., and R. James Purser. 2006. Fibonacci grids: A novel approach to global modelling. *Q. J. R. Meteorol. Soc.* 132:1769–1793. https://doi.org/10.1256/qj.05.227.

33. Cherry, J., A. E. Lindsay, ..., B. Quaife. 2022. Trapping of Planar Brownian Motion: Full First Passage Time Distributions by Kinetic Monte Carlo, Asymptotic, and Boundary Integral Methods. *Multiscale Model. Simul.* 20:1284–1314. https://doi.org/10.1137/21M146380X.

34. Bernoff, A. J., A. E. Lindsay, and D. D. Schmidt. 2018. Boundary Homogenization and Capture Time Distributions of Semipermeable Membranes with Periodic Patterns of Reactive Sites. *Multiscale Model. Simul.* 16:1411–1447.

35. Bruna, M., S. J. Chapman, and G. Z. Ramon. 2015. The effective flux through a thin-film composite membrane. *Europhys. Lett.* 110, 40005. http://stacks.iop.org/0295-5075/110/i=4/a=40005.

36. Handy, G., and S. D. Lawley. 2021. Revising Berg-Purcell for finite receptor kinetics. *Biophys. J.* 120:2237–2248.

37. Berezhkovskii, A. M., M. I. Monine, ..., S. Y. Shvartsman. 2006. Homogenization of boundary conditions for surfaces with regular arrays of traps. *J. Chem. Phys.* 124, 036103.

38. Lindsay, A. E., A. J. Bernoff, and M. J. Ward. 2017. First Passage Statistics for the Capture of a Brownian Particle by a Structured Spherical Target with Multiple Surface Traps. *Multiscale Model. Simul.* 15:74–109.

39. Muratov, C. B., and S. Y. Shvartsman. 2008. Boundary homogenization for periodic array of absorbers. *Multiscale Model. Simul.* 7:44–61.

40. Pillay, S., M. J. Ward, ..., T. Kolokolnikov. 2010. An Asymptotic Analysis of the Mean First Passage Time for Narrow Escape Problems: Part I: Two-Dimensional Domains. *Multiscale Model. Simul.* 8:803–835.

41. Lindsay, A. E., T. Kolokolnikov, and J. C. Tzou. 2015. Narrow escape problem with a mixed trap and the effect of orientation. *Phys. Rev. E*. 91, 032111.

42. Bernoff, A. J., and A. E. Lindsay. 2018. Numerical approximation of diffusive capture rates by planar and spherical surfaces with absorbing pores. *SIAM J. Appl. Math.* 78:266–290.

43. Kaye, J., and L. Greengard. 2020. A fast solver for the narrow capture and narrow escape problems in the sphere. *J. Comput. Phys. X*. 5, 100047.

44. Chou, C.-S., L. Bardwell, T.-M. Yi..., 2011. Noise filtering tradeoffs in spatial gradient sensing and cell polarization response. *BMC Syst. Biol.* 5:196.

45. Ventura, A. C., A. Bush, ..., A. Colman-Lerner. 2014. Utilization of extracellular information before ligand-receptor binding reaches equilibrium expands and shifts the input dynamic range. *Proc. Natl. Acad. Sci. USA*. 111:E3860–E3869.

46. Janetopoulos, C., T. Jin, and P. Devreotes. 2001. Receptor-mediated activation of heterotrimeric G-proteins in living cells. *Science*. 291:2408–2411.

47. Servant, G., O. D. Weiner, ..., H. R. Bourne. 2000. Polarization of chemoattractant receptor signaling during neutrophil chemotaxis. *Science*. 287:1037–1040.

48. Yi, T.-M., H. Kitano, and M. I. Simon. 2003. A quantitative characterization of the yeast heterotrimeric G protein cycle. *Proc. Natl. Acad. Sci. USA*. 100:10764–10769.

49. Perelson, A. S., and G. Weisbuch. 1997. Immunology for physicists. *Rev. Mod. Phys.* 69:1219–1268. https://doi.org/10.1103/RevModPhys.69.1219.

50. Lakhani, V., and T. C. Elston. 2017. Testing the limits of gradient sensing. *PLoS Comput. Biol.* 13:e1005386.

51. Hegemann, B., M. Unger, ..., M. Peter. 2015. A cellular system for spatial signal decoding in chemical gradients. *Dev. Cell*. 35:458–470.

52. Wang, X., W. Tian, ..., D. E. Stone. 2019. Mating yeast cells use an intrinsic polarity site to assemble a pheromone-gradient tracking machine. *J. Cell Biol.* 218:3730–3752.

53. Rappel, W.-J., P. J. Thomas, …, W. F. Loomis. 2002. Establishing direction during chemotaxis in eukaryotic cells. *Biophys. J.* 83:1361–1367.

54. Jilkine, A., and L. Edelstein-Keshet. 2011. A comparison of mathematical models for polarization of single eukaryotic cells in response to guided cues. *PLoS Comput. Biol.* 7, e1001121.

55. Mori, Y., A. Jilkine, and L. Edelstein-Keshet. 2008. Wave-pinning and cell polarity from a bistable reaction-diffusion system. *Biophys. J.* 94:3684–3697.

56. Xiong, Y., C.-H. Huang, …, P. N. Devreotes. 2010. Cells navigate with a local-excitation, global-inhibition-biased excitable network. *Proc. Natl. Acad. Sci. USA.* 107:17079–17086.

57. Jilkine, A., S. B. Angenent, …, S. J. Altschuler. 2011. A density-dependent switch drives stochastic clustering and polarization of signaling molecules. *PLoS Comput. Biol.* 7, e1002271.

58. Kolokolnikov, T., and A. Lindsay. 2015. Recovering multiple small inclusions in a three-dimensional domain using a single measurement. *Inverse Probl. Sci. Eng.* 23:377–388.

59. Chen, W., Q. Nie, …, C.-S. Chou. 2016. Modelling of yeast mating reveals robustness strategies for cell-cell interactions. *PLoS Comput. Biol.* 12, e1004988.

60. Mugler, A., A. Levchenko, and I. Nemenman. 2016. Limits to the precision of gradient sensing with spatial communication and temporal integration. *Proc. Natl. Acad. Sci. USA.* 113:E689–E695.

61. Ellison, D., A. Mugler, …, A. Levchenko. 2016. Cell–cell communication enhances the capacity of cell ensembles to sense shallow gradients during morphogenesis. *Proc. Natl. Acad. Sci. USA.* 113:E679–E688.

62. Camley, B. A., J. Zimmermann, …, W. J. Rappel. 2016. Collective Signal Processing in Cluster Chemotaxis: Roles of Adaptation, Amplification, and Co-attraction in Collective Guidance. *PLoS Comput. Biol.* 12:e1005008.

63. Camley, B. A. 2018. Collective gradient sensing and chemotaxis: modeling and recent developments. *J. Phys. Condens. Matter.* 30, 223001.

64. Lander, A. D. 2013. How cells know where they are. *Science.* 339:923–927.

65. Wolpert, L. 1969. Positional information and the spatial pattern of cellular differentiation. *J. Theor. Biol.* 25:1–47.

66. Gregor, T., D. W. Tank, …, W. Bialek. 2007. Probing the limits to positional information. *Cell.* 130:153–164.

67. Bergmann, S., O. Sandler, …, N. Barkai. 2007. Pre-steady-state decoding of the Bicoid morphogen gradient. *PLoS Biol.* 5:e46.

68. Saunders, T., and M. Howard. 2009. When it pays to rush: interpreting morphogen gradients prior to steady-state. *Phys. Biol.* 6, 046020.

69. Kutejova, E., J. Briscoe, and A. Kicheva. 2009. Temporal dynamics of patterning by morphogen gradients. *Curr. Opin. Genet. Dev.* 19:315–322.

70. Richards, D. M., and T. E. Saunders. 2015. Spatiotemporal Analysis of Different Mechanisms for Interpreting Morphogen Gradients. *Biophys. J.* 108:2061–2073. https://www.sciencedirect.com/science/article/pii/S0006349515002751.

Biophysical Journal, Volume *122*

Supplemental information

Single-cell directional sensing from just a few receptor binding events

Andrew J. Bernoff, Alexandra Jilkine, Adrián Navarro Hernández, and Alan E. Lindsay

Manuscript submitted to **Biophysical** *Journal*
Article

Supplemental material to Single cell directional sensing from just a few receptor binding events.

Andrew J. Bernoff[a], Alexandra Jilkine[b], Adrián Navarro Hernández[b], and Alan E. Lindsay[b]

[a]Department of Mathematics, Harvey Mudd College, CA, 91711, USA.
[b]Department of Applied & Computational Mathematics & Statistics, University of Notre Dame, South Bend, IN, 46556, USA
[*]Correspondence: a.lindsay@nd.edu

1 SPHERICAL ARRIVAL: HOMOGENIZED BC

We wish to solve for the spherical arrival time $p(r,\theta,t)$ which satisfies the axisymmetric three dimensional diffusion problem

$$\frac{\partial p}{\partial t} = D\left[\frac{1}{r^2}\frac{\partial}{\partial r}\left(r^2\frac{\partial p}{\partial r}\right) + \frac{1}{r^2\sin\theta}\frac{\partial}{\partial\theta}\left(\sin\theta\frac{\partial p}{\partial\theta}\right)\right], \qquad r>1, \quad \theta\in(0,\pi) \tag{1a}$$

$$Dp_r(1,\theta,t) = \kappa p(1,\theta,t), \qquad \theta\in(0,\pi); \tag{1b}$$

$$p(r,\theta,0) = \frac{1}{2\pi R^2\sin\theta}\delta(r-R)\delta(\theta), \qquad r>1, \quad \theta\in(0,\pi), \tag{1c}$$

for which we wish to compute the surface flux density $\mathcal{J}(\theta,t) = Dp_r(1,\theta,t)$. A solution to (1) is sought via the Laplace transform, $\hat{p}(r,\theta) = \int_0^\infty e^{-st}p(r,\theta,t)dt$ where $\hat{p}(r,\theta)$ solves the modified Helmholtz equation,

$$D\left[\frac{1}{r^2}\frac{\partial}{\partial r}\left(r^2\frac{\partial \hat{p}}{\partial r}\right) + \frac{1}{r^2\sin\theta}\frac{\partial}{\partial\theta}\left(\sin\theta\frac{\partial \hat{p}}{\partial\theta}\right)\right] - s\hat{p} = \frac{-1}{2\pi R^2\sin\theta}\delta(r-R)\delta(\theta) \quad r>1, \quad \theta\in(0,\pi); \tag{2a}$$

$$D\hat{p}_r(1,\theta) = \kappa\hat{p}(1,\theta), \quad \theta\in(0,\pi). \tag{2b}$$

for which the Laplace transform of the surface flux density satisfies

$$\widehat{\mathcal{J}}(\theta,s) = \int_0^\infty e^{-st}\left[Dp_r(1,\theta,t)\right]dt = D\hat{p}_r(1,\theta).$$

We construct a separable series solution of (2) which is finite as $r\to\infty$, satisfies $D\hat{p}_r(1,\theta) = \kappa\hat{p}(1,\theta)$, and is continuous at $r=R$,

$$\hat{p}(r,\theta) = \begin{cases} \displaystyle\sum_{n=0}^{\infty} A_n\left[i_n(cr) - \frac{cDi_n'(c)-\kappa i_n(c)}{cDk_n'(c)-\kappa k_n(c)}k_n(cr)\right]P_n(\cos\theta), & r<R; \\ \displaystyle\sum_{n=0}^{\infty} A_n\left[\frac{i_n(cR)}{k_n(cR)} - \frac{cDi_n'(c)-\kappa i_n(c)}{cDk_n'(c)-\kappa k_n(c)}\right]k_n(cr)P_n(\cos\theta), & r>R, \end{cases} \tag{3}$$

where $c=\sqrt{s/D}$ and $k_n'(x)$, $i_n'(x)$ are derivatives of the modified spherical Bessel functions. The constants A_n are fixed by incorporating the Dirac source on the right hand side of (2a). The jump condition is

$$2\pi cDR^2A_n\left[\frac{i_n(cR)}{k_n(cR)}k_n'(cR) - i_n'(cR)\right] = -\frac{(2n+1)}{2}. \tag{4}$$

Solving yields

$$A_n = \frac{1}{2\pi D}\frac{(2n+1)}{2}c\,k_n(cR),$$

where we have used the Wronskian identity $i_n'(z)k_n(z) - i_n(z)k_n'(z) = z^{-2}$. Now returning to $c = \sqrt{s/D}$, the Laplace transform of flux through the spherical surface is then

$$\begin{aligned}
\widehat{\mathcal{J}}(\theta, s) &= D\hat{p}_r(1, \theta, s) \\
&= cD \sum_{n=0}^{\infty} A_n \left[i_n'(c) - \frac{cDi_n'(c) - \kappa\, i_n(c)}{cDk_n'(c) - \kappa\, k_n(c)} k_n'(c) \right] P_n(\cos\theta), \\
&= \frac{1}{2\pi} \sum_{n=0}^{\infty} \frac{(2n+1)}{2} \left[\frac{k_n(cR)/k_n'(c)}{k_n(c)/k_n'(c) - cD/\kappa} \right] P_n(\cos\theta). \\
&= \frac{1}{2\pi} \sum_{n=0}^{\infty} \frac{(2n+1)}{2} \psi_n(c) P_n(\cos\theta), \qquad \psi_n(c) = \frac{k_n(cR)/k_n'(c)}{k_n(c)/k_n'(c) - cD/\kappa}.
\end{aligned} \tag{5}$$

The Laplace transform of the total flux is

$$\begin{aligned}
\widehat{\rho}(s) &= 2\pi \int_0^{\pi} \widehat{\mathcal{J}}(\theta, s)\, \sin\theta\, d\theta \\
&= \int_0^{\pi} \sum_{n=0}^{\infty} \frac{(2n+1)}{2} \psi_n(c) P_n(\cos\theta)\, \sin\theta\, d\theta \\
&= \sum_{n=0}^{\infty} \frac{(2n+1)}{2} \psi_n(c) \int_{-1}^{1} P_n(x)\, dx \\
&= \psi_0(c) = \frac{k_0(cR)/k_0'(c)}{k_0(c)/k_0'(c) - cD/\kappa} = \frac{e^{-(R-1)\sqrt{s/D}}}{R[1 + (D/\kappa)\cdot(1 + \sqrt{s/D})]},
\end{aligned}$$

where we've used the zero mean property of the Legendre polynomials. The inverse Laplace transform of this quantity is the distribution of arrival times at the sphere,

$$\rho(t) = \frac{\kappa}{R} e^{-\frac{(R-1)^2}{4Dt}} \left[\frac{1}{\sqrt{\pi D t}} - \mathrm{erfc}(\beta)\, e^{\beta^2}\, (\kappa/D + 1) \right]. \tag{6}$$

where $\beta = \frac{R-1}{2\sqrt{Dt}} + (\kappa/D + 1)\sqrt{Dt}$.

The CDF of this distribution can then be calculated as

$$F(t) = \int_0^t \rho(\tau)\, d\tau = \frac{1}{(1 + D/\kappa)\, R} \left[\mathrm{erfc}\left(\frac{R-1}{2\sqrt{Dt}} \right) - \mathrm{erfc}(\beta)\, e^{\beta^2} e^{-\frac{(R-1)^2}{4Dt}} \right]. \tag{7}$$

We remark that

$$\int_0^{\infty} \rho(t)\, dt = \lim_{t\to\infty} F(t) = \frac{1}{(1 + D/\kappa)R}$$

so that the probability of capture is not unity, but inversely proportional to the initial distance to the sphere.

1.1 Short time asymptotics via the method of moments

In this section we obtain the short-time asymptotics for the solution of (1) which will be used to describe the source detection for very early arrivals to the cellular surface. This is a familiar problem from stochastic processes; the earliest arrivals are concentrated at the point closest to the source. Heuristically, this can be viewed as a boundary layer calculation. The outer solution is just the free space Green's function and the inner solution is confined to a boundary layer of width $\sqrt{Dt}$ at the edge of the sphere. Specifically, we will consider the problem when the diffusion length is much longer than the typical receptor size and spacing (so the homogenization approximation is valid) but much smaller that the sphere radius. We expect that the arrivals will be concentrated near the point on the sphere closest to the source, here taken as the polar axis (where $\theta = 0$).

The homogenized problem derived above allows a straightforward characterization of the fluxes at short times via the method of moments. Our starting point is the expansion of the Laplace transform of the flux density (5) as a Legendre series (reflecting the axisymmetry of the distribution). From the orthogonality of the Legendre modes,

$$\psi_n(c) = 2\pi \int_{\theta=0}^{\pi} \widehat{\mathcal{J}}(\theta, s) P_n(\cos\theta) \sin\theta\, d\theta, \tag{8}$$

where $c = \sqrt{s/D}$. Next we exploit the exponential localization of the distribution to treat the interval $\theta \in [0, \pi]$ as effectively infinite. We define the moments of a radially symmetric two-dimensional distribution, $\mathcal{J}(\theta, t)$ as $M_n(t)$ and their Laplace transform $\widehat{M}_n(s) = \mathcal{L}[M_n(t)]$,

$$M_n(t) = 2\pi \int_{\theta=0}^{\pi} \mathcal{J}(\theta, t)\, \theta^n \, \sin\theta d\theta, \qquad \widehat{M}_n(s) = 2\pi \int_{\theta=0}^{\pi} \widehat{\mathcal{J}}(\theta, s)\, \theta^n \, \sin\theta d\theta. \tag{9}$$

The linearity of the moments implies that the Laplace transform of the moments are the moments of the Laplace transform. The axisymmetry implies the mean and the skewness of the distribution vanish and that the radial moments of interest are even n.

The zeroth moment is exactly $\psi_0(s)$

$$\widehat{M}_0(s) = \psi_0(c) = \frac{k_0(cR)/k_0'(c)}{k_0(c)/k_0'(c) - cD/\kappa} = \frac{e^{-(R-1)\sqrt{s/D}}}{R[1 + (D/\kappa)\cdot(1 + \sqrt{s/D})]},$$

whose inverse transform we have computed above

$$M_0(t) = \frac{\kappa}{R} e^{-\frac{(R-1)^2}{4Dt}} \left[\frac{1}{\sqrt{\pi D t}} - \operatorname{erfc}(\beta)\, e^{\beta^2} \left(\kappa/D + 1\right) \right], \tag{10}$$

where $\beta = \frac{R-1}{2\sqrt{Dt}} + (\kappa/D + 1)\sqrt{Dt}$. Assuming $t \ll 1$ which implies $\beta \gg 1$ and allowing that κ/D may be large yields the uniform approximation that

$$M_0(t) = \frac{\kappa}{R} \frac{e^{-\frac{(R-1)^2}{4Dt}}}{\sqrt{\pi D t}} \left[\frac{R-1}{(R-1) + 2\kappa t} \right] [1 + O(Dt)] \,.$$

To compute the higher moments, we make the ansatz that $\widehat{M}_{2k}(s) \sim \widehat{M}_0(s) c^{-2k}$. Expanding the first few Legendre polynomials near $\theta = 0$ yields

$$P_0(\cos\theta) = 1, \quad P_1(\cos\theta) = \cos\theta = 1 - \frac{1}{2}\theta^2 + \frac{1}{24}\theta^4 + \cdots, \quad P_2(\cos\theta) = \frac{3}{2}\cos^2\theta - \frac{1}{2} = 1 - \frac{3}{2}\theta^2 + \frac{1}{2}\theta^4 + \cdots$$

which yields

$$\widehat{M}_2(c) \sim -2\left[\chi_1(c) - \chi_0(c)\right] \sim 2\frac{R-1}{R^2} \frac{e^{-(R-1)\sqrt{s/D}}}{\sqrt{s/D}[1 + (D/\kappa)\sqrt{s/D})]} \cdot \left[1 + O\left(\frac{1}{\sqrt{s/D})} \right) \right],$$

whose inverse transform can be approximated for $\alpha, \beta \gg 1$ as

$$M_2(t) = M_0(t) \left[\frac{4Dt}{R} \right] \cdot \left[1 + O(\sqrt{Dt}) \right].$$

This allows us to compute the variance

$$\operatorname{Var}[\mathcal{J}(\theta, t)] \equiv \frac{M_2(t)}{M_0(t)} \sim \frac{4Dt}{R}.$$

A similar tedious calculation yields the result that

$$M_4(t) = M_0(t) \left[\frac{32(Dt)^2}{R^2} \right] \cdot \left[1 + O(\sqrt{Dt}) \right],$$

and shows that the kurtosis satisfies

$$\operatorname{Kur}[\mathcal{J}(\theta, t)] \equiv \frac{M_4(t)\cdot M_0(t)}{[M_2(t)]^2} \sim 2 + O(\sqrt{Dt}).$$

This is consistent with a Gaussian distribution, specifically, we have in the limit $t \longrightarrow 0^+$ that

$$\mathcal{J}(\theta, t) \sim \frac{\mathcal{M}}{2\pi\sigma^2} e^{-\frac{\theta^2}{2\sigma^2}}; \qquad \sigma^2 = \frac{1}{2} \operatorname{Var}[\mathcal{J}(\theta, t)] \sim \frac{2Dt}{R}; \tag{11a}$$

$$\mathcal{M} = M_0(t) = \frac{\kappa}{R} \frac{\mathrm{e}^{-\frac{(R-1)^2}{4Dt}}}{\sqrt{\pi D t}} \left[\frac{R-1}{(R-1) + 2\kappa t} \right] [1 + O(Dt)] \,. \tag{11b}$$

Finally, if we are measuring a density, $J(\theta, t)$ that is solely a measure of the polar angle we need to integrate out the azimuthal component of the area element $dA = 2\pi \sin\theta \, d\theta$ which yields

$$J(\theta, t) = 2\pi \sin\theta \cdot \mathcal{J}(\theta, t) \sim 2\pi\theta\mathcal{J}(\theta, t) = \mathcal{M}\frac{\theta}{\sigma^2}e^{-\frac{\theta^2}{2\sigma^2}}.$$

In terms of the elevation coordinate $z = \cos\theta$, the transformed density is

$$J(z, t) = \frac{J(\theta, t)}{|dz/d\theta|} = \frac{\mathcal{M}}{\sigma^2}e^{-\frac{1-z}{\sigma^2}},$$

where we have used $\frac{\theta^2}{2} = 1 - z + O(\theta^4)$ for $\theta \ll 1$.

1.2 Asymptotics of scaling and centering parameters.

Here we develop and asymptotic solution to the nonlinear equation

$$h(t)e^{-\frac{(R-1)^2}{4Dt}} = M^{-1}, \tag{12a}$$

$$h(t) = A\frac{t^{\frac{3}{2}}}{(1+Bt)}, \qquad A = \sqrt{\frac{D}{\pi}}\frac{4\kappa}{R(R-1)^2}, \qquad B = \frac{2\kappa}{R-1}, \tag{12b}$$

as $M \longrightarrow \infty$. We introduce the following rescaling of (12)

$$z^{-1}e^{-z}(1+\alpha z^{-1})^{-\frac{2}{3}} = \varepsilon, \qquad z = \frac{(R-1)^2}{6Dt}; \tag{13a}$$

$$\varepsilon = \frac{6D}{(R-1)^2}(MA)^{-\frac{2}{3}} = \left[\sqrt{\frac{27\pi}{2}}\frac{RD}{M\kappa(R-1)}\right]^{\frac{2}{3}}, \qquad \alpha = \frac{(R-1)^2}{6D}B = \frac{\kappa(R-1)}{3D} \tag{13b}$$

and solve (13) in the limit $\varepsilon \longrightarrow 0$. After taking logarithms of (13a), we obtain an equation of form $z = f(z)$ and define the iterative scheme

$$z_{n+1} = f(z_n), \qquad f(z) = -\log\varepsilon - \log z - \frac{2}{3}\log\left(1+\alpha z^{-1}\right). \tag{14a}$$

Taking $\nu = -\log\varepsilon$, the first three iterations as $\nu \longrightarrow \infty$ are

$$z_1 = \nu \tag{14b}$$

$$z_2 = f(z_1) = \nu - \log\nu - \frac{2}{3}\log\left(1+\frac{\alpha}{\nu}\right) \sim \nu - \log\nu - \frac{2\alpha}{3\nu} + O(\nu^{-2}) \tag{14c}$$

$$\begin{aligned} z_3 = f(z_2) &= \nu - \log\left[\nu - \log\nu - \frac{2\alpha}{3\nu}\right] - \frac{2}{3}\log\left(1+\alpha\left[\nu - \log\nu - \frac{2\alpha}{3\nu}\right]^{-1}\right) \\ &\sim \nu - \log\nu + \frac{\log\nu}{\nu} - \frac{2\alpha}{3\nu} + O\left(\frac{\log\nu}{\nu^2}\right). \end{aligned} \tag{14d}$$

From (13b), we therefore have the rescaled solution

$$t = \frac{(R-1)^2}{6Dz} = \frac{(R-1)^2}{6D\nu}\left[1 - \frac{\log\nu}{\nu} + \frac{\log\nu}{\nu^2} - \frac{2\alpha}{3\nu^2} + O\left(\frac{\log\nu}{\nu^3}\right)\right]^{-1}. \tag{15}$$

1.3 Optimal number of binding events.

In this section we explore the possibility for an optimizing number of binding events. We concentrate on characterizing the average elevation of the first K arrivals given by the variable $Z_K = \frac{1}{K}\sum_{i=1}^{K} z_i$. This variable, arising from a sum of exponential variables, follows a hypoexponential distribution which allows for a succinct analysis of its properties.

The value $Z_K = 1$ is the true source and we determine that the mean error satisfies

$$\mathbb{E}[1 - Z_K] = \sum_{i=1}^{K} \frac{\lambda_i}{K} = \frac{2D}{R}\Big(b_M + \frac{a_M}{K}\log K!\Big),$$

while the variance in the error is given by

$$\mathrm{Var}[1 - Z_K] = \sum_{i=1}^{K} \frac{\lambda_i^2}{K^2} = \frac{4D^2}{R^2K^2}\sum_{i=1}^{K}\Big(b_M + a_M \log i\Big)^2.$$

The errors increase in mean and decrease in variance as K increases. Can a balance of these trade-offs results in an optimal K? To explore this, we defined confidence intervals U_c for the hitting region $Z_K \in (z_c(K), 1)$ such that $\mathbb{P}[z_c(K) < Z_K < 1] = U_c$. Here the CDF $F_{Z_K}(z) = \mathbb{P}[z < Z_K < 1]$ is given by

$$F_{Z_K}(z) = \int_z^1 \mathbb{P}[Z_K = z']dz' = \sum_{i=1}^{K} w_i(1 - e^{-K(1-z)/\lambda_i}). \tag{16}$$

For several values of $U_c \in (0, 1)$, we obtained curves of $z_c(K)$ against K which are relatively flat but yield an optimizing value (Fig. 1). The flatness in of $z_c(K)$ is due to the slight decrease in $\mathbb{E}[1 - Z_K]$ as K increases, meaning that the extent of the hitting region is relatively insensitive to K. We observe the existence of an optimizing K only for $U_c \gtrapprox 0.77$. For such values of U_c where optimizing values of K exist, we find that these critical points depend quite sensitively on K. However, we do observe that only around $K \approx 5$ binding events are necessary to bring $z_c(K)$ quite close to the optimal value.

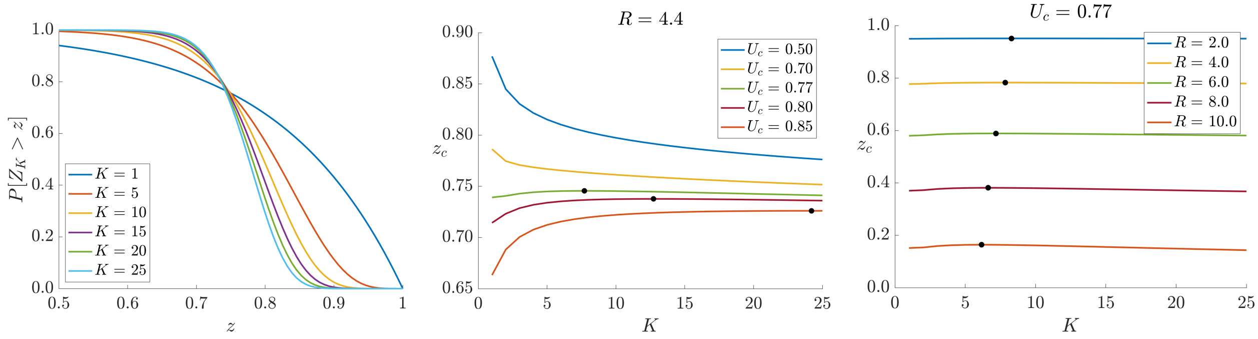

(a) $F_{Z_K}(z)$ for various K. (b) $z_c(K)$ for various U_c. (c) $z_c(K)$ for various R.

Figure 1: Exploration of optimizing number of binding events K. Left: The CDF $F_{Z_K}(z)$ for various increasing values of K. Center: Left boundary $z_c(K)$ of the hitting region $Z_K \in (z_c(K), 1)$ for various confidence intervals U_c and fixed value $R = 4.4$. Right: Plots of $z_c(K)$ for various R and fixed confidence interval $U_c = 0.77$.
